# Comparative Transcriptome Profiling of Skeletal Muscle from Black Muscovy Duck at Different Growth Stages Using RNA-seq

**DOI:** 10.3390/genes11101228

**Published:** 2020-10-20

**Authors:** Zhigang Hu, Junting Cao, Guangyu Liu, Huilin Zhang, Xiaolin Liu

**Affiliations:** College of Animal Science and Technology, Northwest A&F University, Yangling 712100, China; huzhigang2017060163@nwafu.edu.cn (Z.H.); fightingcaoting@nwafu.edu.cn (J.C.); guangyuliu@nwafu.edu.cn (G.L.); zhhl7461@nwsuaf.edu.cn (H.Z.)

**Keywords:** skeletal muscle, transcriptome, gene expression, duck, growth stage

## Abstract

In China, the production for duck meat is second only to that of chicken, and the demand for duck meat is also increasing. However, there is still unclear on the internal mechanism of regulating skeletal muscle growth and development in duck. This study aimed to identity candidate genes related to growth of duck skeletal muscle and explore the potential regulatory mechanism. RNA-seq technology was used to compare the transcriptome of skeletal muscles in black Muscovy ducks at different developmental stages (day 17, 21, 27, 31, and 34 of embryos and postnatal 6-month-olds). The SNPs and InDels of black Muscovy ducks at different growth stages were mainly in “INTRON”, “SYNONYMOUS_CODING”, “UTR_3_PRIME”, and “DOWNSTREAM”. The average number of AS in each sample was 37,267, mainly concentrated in TSS and TTS. Besides, a total of 19 to 5377 DEGs were detected in each pairwise comparison. Functional analysis showed that the DEGs were mainly involved in the processes of cell growth, muscle development, and cellular activities (junction, migration, assembly, differentiation, and proliferation). Many of DEGs were well known to be related to growth of skeletal muscle in black Muscovy duck, such as *Myo*G, *FBXO*1, *MEF2A,* and *FoxN*2. KEGG pathway analysis identified that the DEGs were significantly enriched in the pathways related to the focal adhesion, MAPK signaling pathway and regulation of the actin cytoskeleton. Some DEGs assigned to these pathways were potential candidate genes inducing the difference in muscle growth among the developmental stages, such as *FAF*1, *RGS*8, *GRB*10, *SMYD*3, and *TNNI*2. Our study identified several genes and pathways that may participate in the regulation of skeletal muscle growth in black Muscovy duck. These results should serve as an important resource revealing the molecular basis of muscle growth and development in duck.

## 1. Introduction

Skeletal muscle is the largest and most important tissue in animals, accounting for about 50% of body weight [1,2]. It participates in body movement, protection, and metabolic regulation [3]. Because meat yield directly determines the level of economic benefits, the study of skeletal muscle development is important in animal husbandry production. The growth and development of duck skeletal muscle is an essential economic trait, which is determined by genetic, and influenced by nutritional and environmental factors. The development of embryonic skeletal muscle is a tightly regulated process that is critically modulated by genes and related signaling pathways [4,5]. During prenatal and very early postnatal development, muscle growth in vertebrates depends on an increasing number of muscle fibers (hyperplasia) [6,7]. After postnatal growth, the increase in skeletal muscle is mainly due to muscle hypertrophy, accompanied by the proliferation of satellite cells and then the new myonuclei is incorporated into existing myofibers [8]. Black Muscovy duck, an excellent meat duck breed in China, has the advantages of fast growth and high lean meat rate. Most notably, few studies have investigated the mechanisms regulating growth patterns of skeletal muscle in black Muscovy duck. Since the growth of skeletal muscle is controlled by multiple genes, a more systematic understanding of the genes expressed at different growth stages in black Muscovy duck is needed.

Transcriptome sequencing (RNA-seq) is a technology that analyzing the transcripts by deep sequencing technology, which detects the whole transcriptome at the single nucleotide level [9]. It analyzes the structure and expression level of transcripts, which is an important method of gene expression and transcription analysis [10]. In recent years, RNA-seq has been widely used in study on livestock and poultry transcriptomes. Compared with other gene expression profiling methods, RNA-Seq has advantages in detecting mRNA expression in different tissues or at different developmental stages, which is helpful to reveal new genes and splicing variations [11], as well as the pathways [12]. Several studies at mRNA level have been performed in poultry birds, with the aims to investigate and analyze the genes and pathways that influence the growth and development of skeletal muscle. Xue et al. (2018) compared the mRNA expression of leg muscles of Jinghai Yellow Chickens at early growth stages by RNA-seq, a total of 978 differentially expressed genes (DEGs) were identified, and five significantly enriched pathways were found: EMC–receptor interaction, focal adhesion, tight junction, insulin signaling pathway, and regulation of the actin cytoskeleton [13]. Transcriptome analysis of breast muscle in Commercial Layers of Roman, White Broiler, and Daheng chickens showed that genes related to positive cell proliferation, growth, cell differentiation and developmental processes were more enriched, and the pathways, including ECM–receptor interaction, MAPK signaling pathway and focal adhesion, were the most enriched DEGs [14]. Xu et al. (2017) analyzed the gene expression profiles of Pekin ducks during incubation period, and the results showed that the DEGs related to cell division (M phase of mitotic cell cycle, cell division, mitosis, and mitotic prometaphase), and the pathways, including DNA replication, Cell cycle, Gap junction, were significantly enriched at hatched day 19 [15]. Zhu et al. (2017) analyzed potential candidate genes and signaling pathways related to the development of breast muscle during late-term embryonic to neonatal development, a total of 393 DEGs were identified, and the DEGs involved in different metabolism pathways (such as metabolic pathways, citrate cycle, glycine, serine, and threonine metabolism, sulfur metabolism, carbon metabolism, and pyruvate metabolism) [16].

The purpose of this study was to investigate the major DEGs and their expression pathways in skeletal muscle of black Muscovy duck by using RNA-Seq technology and bioinformatic tools. We found the genes and molecular mechanisms involved in this developmental process by carrying out a comprehensive analysis of genes with expression levels that reflected the growth pattern of breast and leg muscles in black Muscovy duck. Our findings are useful for understanding the mechanisms regulating the development of skeletal muscle and the pattern of duck growth.

## 2. Materials and Methods

### 2.1. Animal and Muscle Tissue Collection

One hundred eggs of black Muscovy duck were incubated in a standard incubator after disinfection. A total of 8 eggs were sampled from the day 17 (BE17), day 21 (BE21), day 27 (BE27), day 31 (BE31), and day 34 (BE34) of the incubation period, and the breast and leg muscle were separated for DNA and RNA extraction. DNA of muscle tissue was extracted according to the phenol chloroform protocol. According to the sequence of chromatin helix DNA binding protein 1 (CHD1) gene on sex chromosomes of duck, sex identification primers (gCHD) were used [17] and female embryos were selected as the research objects (Table 1), because the female Muscovy duck accounted for the majority of duck farms due to the reason of laying eggs, and the same gender can avoid the error of sequencing data. Besides, 6-month-old female ducks (BM6), raising under the same environmental conditions and free access to feed and water (Table 2), were slaughtered quickly to collect the breast muscle and leg muscle. Muscles were excised and immediately frozen in liquid nitrogen and stored at −80 °C until further use. Animal care, slaughter and experimental procedures were approved by Institutional Animal Care and Institutional Ethic Committee of Northwest A&F University (ethic code: #0326/2019).

### 2.2. RNA Extraction, Library Construction and Sequencing

The total RNA was extracted from the breast and leg muscle separately using QIAzol Lysis Reagent according to the manufacturer’s instructions (QIAGEN, BerlinCity Germany). The RNA integrity was measured using a 2100 Bioanalyzer (Agilent Technologies, San JoseCity USA), the RNA purity and concentration were verified by agarose gel electrophoresis and Nanodrop 2000 (Thermo, Waltham, CA, USA).

Thirty-six sequencing libraries were construct by the TruSeq PE Cluster Kit v4-cBot-HS (Illumina HiSeq X Ten platform, San Diego, CA, USA) according to the manufacturer’s instructions, and the indexed samples were clustered by the Illumina’s cBot cluster generation system following the manufacturer’s instructions. After cluster generation, the libraries were sequenced on the Illumina platform (Illumina HiSeq X Ten platform, San Diego, CA, USA), and a paired end read of 150 bp was generated.

### 2.3. Sequencing Analysis

After sequencing, raw reads were filtered: (1) Removing reads containing adapters or poly-N; (2) removing reads containing more than 10% of unknown nucleotides; and (3) removing low-quality reads containing more than 50% of low-quality (Q-value ≤ 10) bases. Besides, quality parameters for filtered data including Q30, GC content, and sequence-duplication level were used for data filtering. All downstream analyses were based on high-quality clean data. The filtered reads were mapped to the *Anas platyrhynchos* (Ap) genome sequence (https://www.ncbi.nlm.nih.gov/genome/?term=DUCK) and annotated transcripts (https://www.ncbi.nlm.nih.gov/assembly/GCF_003850225.1). Based on the Ap genome, further analyses and annotation have been carried out for a perfect matched reads or one mismatched reads. Then, the HISAT 2 tool software were used to map with the Ap genome.

### 2.4. Analysis of SNP/InDel

According to the results of the HISAT 2 comparison between the reads and the Ap genome sequence of each sample, the GATK software was used to find the single base mismatch between the sequenced sample and the Ap genome, and identify the potential single nucleotide polymorphism (SNP) site. The insertion-deletion (InDel) of the sample was also detected by the GATK software. According to the position of heterotopia in the Ap genome, the region where the mutation occurred (intergenic region, gene region or CDS region, etc.) and the effect of mutation (synonymous or non-synonymous mutation, etc.) were got by the SNPEFF software.

### 2.5. Prediction of Variable Splices

The results of HISAT 2 comparison were spliced by the StringTie software. The variable splicing types and corresponding expressions of each sample were obtained by the ASprofile software. The differentially expressed isoforms were estimated by the Cufflink.

### 2.6. Analysis of DEGs

To analyze DEGs, gene coverage and gene expression level were calculated. Gene coverage is the percentage of a gene covered by reads. The unigene expression was calculated and normalized to FPKM (fragments per kilobase per transcript per million mapped reads) based on FPKM (A) = cDNA Fragments/[Mapped Fragments (Millions) Transcript Length (kb)], where cDNA Fragments referred to the number of fragments compared to a transcript; Mapped Fragments (Millions) referred to the total number of fragments compared to a transcript, in 1 × 10^6^ units; Script Length (kb) referred to the length of the transcript, in 1 × 10^3^ bases units.

Differential expression was analyzed using the DESeq 2, and the false discovery rate (FDR) was calculated to calibrate the significant level and eliminate the influence of random fluctuations and errors. The unigenes with Fold change ≥ 2 and FDR < 0.01 were considered DEGs. The Benjamin–Hochberg correction method was used to correct the significant *p*-value of the original hypothesis test, and FDR was used as the key indicator for screening DEGs.

### 2.7. Analysis of GO and KEGG Pathway

GO enrichment analysis was performed using the Goseq R software package, and the enrichment of DEGs in KEGG was tested using the KOBAS software. Besides, gene functions were annotated with the following databases, including Nr (NCBI non-redundant protein sequences, ftp://ftp.ncbi.nih.gov/blast/db/); Nt (NCBI non-redundant nucleotide sequences, ftp://ftp.ncbi.nih.gov/blast/db/); Pfam (Protein family, http://pfam.xfam.org/); KOG/COG (Clusters of Orthologous Groups of proteins, http://www.ncbi.nlm.nih.gov/KOG/; http://www.ncbi.nlm.nih.gov/COG/); Swiss-Prot (a manually annotated and reviewed protein sequence database, http://www.uniprot.org/); GO (Gene Ontology, http://www.geneontology.org/); KO (KEGG Ortholog database, https://www.genome.jp/kegg/ko.html).

### 2.8. RNA-seq Validation by qPCR

In order to confirm the reliability of RNA-seq results, 26 representative responsive genes of breast and leg muscle in black Muscovy duck were selected for qPCR, respectively. Duck *β-actin* (accession number: NC_040060.1) was measured as the housekeeping gene. RNA with an A260/280 ratio between 1.9 and 2.1, and A260/230 ratio > 2.0 were used for cDNA synthesis. The list of primers were described in Table 1. Each reaction mixture contained 5 μL of TransStart Tip Green qPCR SuperMix (Transgen, Beijing, China), 0.8 μL of cDNA (400 ng/μL), 0.3 μL of each primer (10 μM) and 3.6 μL ddH2O in 10 μL final volume using EcoRT48 (OSA, London, UK). The optimal reaction procedure included 95 °C for 30 s, followed by 40 cycles of 95 °C for 5 s, 60 °C for 30 s, then 95 °C for 15 s, 55 °C for 15 s, 95 °C for 15 s. To derive the relative expression value, 2^−△△Ct^ method was adopted. The results were expressed as mean ± SD of at least three independent biological replicates. Statistical analyses of the data were conducted in SPSS 20.0. Significant differences in duck muscle gene expression in different growth periods were determined by one-way ANOVA followed by Tukey’s test and Duncan’s test.

## 3. Results

### 3.1. Transcriptome Profiles

In this study, a total of 36 libraries were established from the breast and leg muscles of duck in BE17, BE21, BE27, BE31, BE34, and BM6. The gene expression of black Muscovy duck skeletal muscle transcriptome at different growth stages was systematically analyzed by high-throughput RNA sequencing. All samples had an RNA integrity number (RIN) > 7.5, and a RNA concentration ≥ 125.2 ng/μL (Table S1). After quality control, clean reads of samples ranging from 19,700,766 to 33,105,790 (24,559,860 on average), GC contents of the samples were between 49.19% and 56.80%, and ≥Q30 (%) were 91.36% to 93.80% (Table 3). In total reads of the samples, over 26,201,608 high-quality reads per sample were mapped to the Ap genome and used for gene expression analysis, where “Uniq Mapped Reads” ranging from 23,077,005 (51.13%) to 30,289,226 (69.98%) and “Multiple Map Reads” ranging from 1,297,423 (2.92%) to 11,929,431 (23.55%). Besides, the number of Reads aligned to the positive strand in the Ap genome were 9,228,720 to 20,801,712 and the number of reads aligned to the negative strand in the Ap genome were between 13,086,584 and 21,955,456 (Table S2).

### 3.2. Annotation and Classification of SNP/InDel

**Figure 1:**
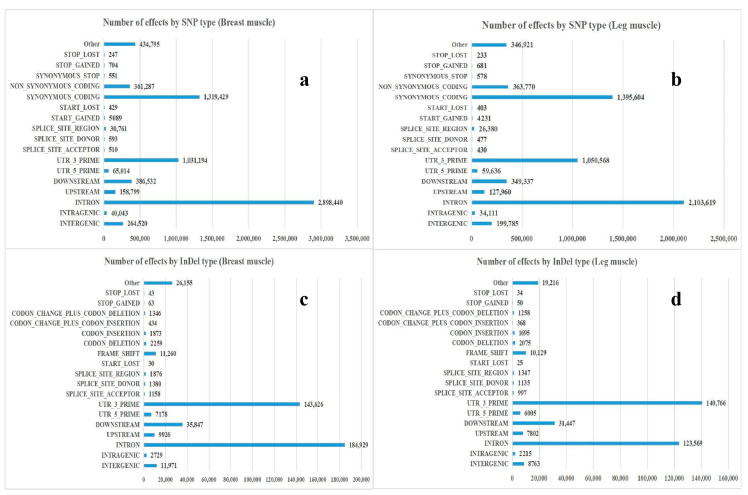
Annotation and classification of SNP/InDel. (**a**) The annotation result of SNP in breast muscle; (**b**) the annotation result of SNP in leg muscle; (**c**) the annotation result of InDel in breast muscle; (**d**) the annotation result of InDel in leg muscle.

The number of SNP loci, the proportion of transition type and transversion type, as well as the ratio of heterozygous SNP loci from each sample were counted, the results were shown in Table 4. There were 23,381 to 634,028 SNPs in skeletal muscle of black Muscovy duck at different growth stages, where the total numbers of SNPs in the genic region were 20,572 to 574,484, the numbers of SNPs between genes were 2809 to 59,544. Besides, the percentage that the transition-type SNP accounts for all SNP locis were over 71.12%, the percentage that the transversion-type SNP loci accounts for all SNP sites were between 23.66% and 28.88%, and the percentage that the heterozygous SNPs account for all SNPs were 4.27% to 48.09%. The annotation results of SNP and InDel were shown in Figure 1. The annotations of SNP were mainly distributed in “INTRON”, “SYNONYMOUS_CODING”, and “UTR_3_PRIME”. The annotations of InDel were mainly distributed in “INTRON”, “UTR_3_PRIME”, and “DOWNSTREAM”.

### 3.3. Prediction of Alternative Splice (AS)

**Figure 2:**
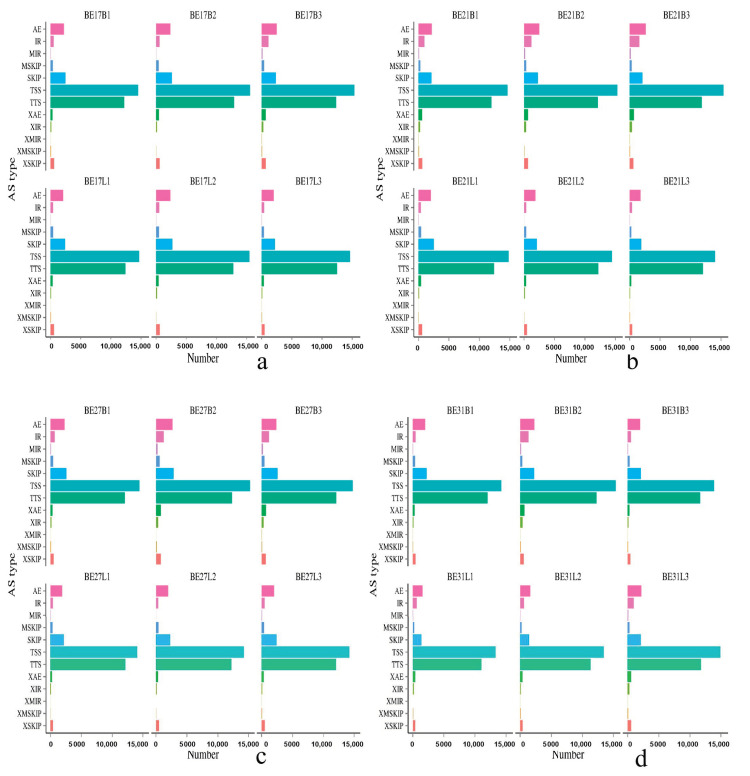
The predicted number of variable splices in black Muscovy ducks during different incubation stages. (**a**–**f**) represent the predicted number of variable splices in black Muscovy ducks on day 17, 21, 27, 31, and 34 of incubation and postnatal 6-month-old, respectively. Note: The horizontal axis represents number to one of alternative transcripts, the vertical axis represents types of alternative splicing events.

The chromosomal position of each transcript was obtained by aligning the sequence to the Ap genome. Twelve different splice patterns in the transcriptome data of duck skeletal muscle were detected: (**A**) TSS: Alternative 5’ first exon (transcription start site) the first exon splicing; (**B**) TTS: Alternative 3’ last exon (transcription terminal site) the last exon splicing; (**C**) SKIP: Skipped exon single exon skipping; (**D**) XSKIP: Approximate SKIP single exon skipping (fuzzy boundary); (**E**) MSKIP: Multi-exon SKIP multi-exon skipping; (**F**) XMSKIP: Approximate MSKIP multi-exon skipping (fuzzy boundary); (**G**) IR: Intron retention single intron retention; (**H**) XIR: Approximate IR single intron retention (fuzzy boundary); (**I**) MIR: Multi-IR multi-intron retention; (**J**) XMIR: Approximate MIR multi-intron retention (fuzzy boundary); (**K**) AE: Alternative exon ends (5’, 3’, or both); (**L**) XAE: Approximate AE variable 5’ or 3’ end (fuzzy boundary). As shown in Figure 2, the average number of AS events per sample was 37,267, and the predicted number of variable splices in each sample were mainly concentrated in TSS and TTS.

### 3.4. Analysis of DEGs

The relative expression of gene was normalized as fragments per kilobase of exon model per million mapped reads (FPKM), which was proportional to the number of cDNA fragments originated by gene transcription. Statistical analysis of DEGs in breast and leg muscle of black Muscovy ducks at different stages were shown in Table 5. In breast muscle, differential gene expression analysis of BE17B_vs_BE21B showed that 410 genes were significantly differentially expressed (Fold change ≥ 2 and FDR < 0.01 at *p* < 0.05, the same below), including 218 up-regulated and 192 down-regulated genes. Moreover, there were 1958 significantly expressed genes from BE17B_vs_BE27B, among which 1162 were up-regulated genes and 796 were down-regulated genes. A number of 1517 DEGs were detected from BE17B_vs_BE31B, the number of up-regulated genes were 925 and the down-regulated genes was 592 respectively. There were 1460 DEGs from BE17B_vs_BE34B, including 852 up-regulated genes and 608 down-regulated genes. Besides, 5377 DEGs were found in BE17B_vs_BM6B, of which 2580 were up-regulated genes and 2797 were down regulated genes (Figure S1).

In leg muscle, the results showed that there were 655 DEGs, including 371 up-regulated genes and 284 down-regulated genes from BE17L_vs_BE21L. Moreover, 2866 DEGs were discovered in BE17L_vs_BE27L, among which 1606 genes were up-regulated and 1260 genes were down-regulated. From BE17L_vs_BE31L, there were 4413 significantly expressed genes, among which 2440 were up-regulated genes and 1973 were down-regulated genes. 4326 DEGs were detected from BE17B_vs_BE34B, out of which 2374 up-regulated and 1952 down-regulated genes were identified as significantly differentially expressed. Besides, 4560 DEGs were discovered in BE17L_vs_BM6L, and 2303 DEGs were up-regulated genes and 2257 DEGs were down-regulated genes (Figure S2).

In the comparison of breast and leg muscle, there were 214, 1256, 195, 1226, 19, and 104 significantly expressed genes from BE17B_vs_BE17L, BE21B_vs_BE21L, BE27B_vs_BE27L, BE31B_vs_BE31L, BE34B_vs_BE34L, and BM6B_vs_BM6L, among which 162, 523, 51, 606, 5, and 58 were up-regulated genes and 52, 733, 144, 620, 14, and 46 were down-regulated genes (Figure S3). The top 5 up- and down-regulated genes between the comparison samples were listed in Table S3. Hierarchical clustering of DEGs showed that the samples clustered based on condition (Figures S4–S6).

### 3.5. Analysis of GO Annotation and KEGG Pathway

Functional annotations of DEGs in the database were performed, and the number of annotated genes were shown in Table S4. The total number of DEGs annotated were between 17 and 5253, where the COG were 3 to 1775, the GO were 14 to 4191 and the KEGG were 5 to 3542. Besides, the number of KOG ranged from 10 to 3788, the NR ranged from 17 to 5231, the Pfam ranged from 14 to 4816. Moreover, the Swiss-Prot were between 12 and 3788, the eggNOG were between 15 and 5118.

**Figure 3:**
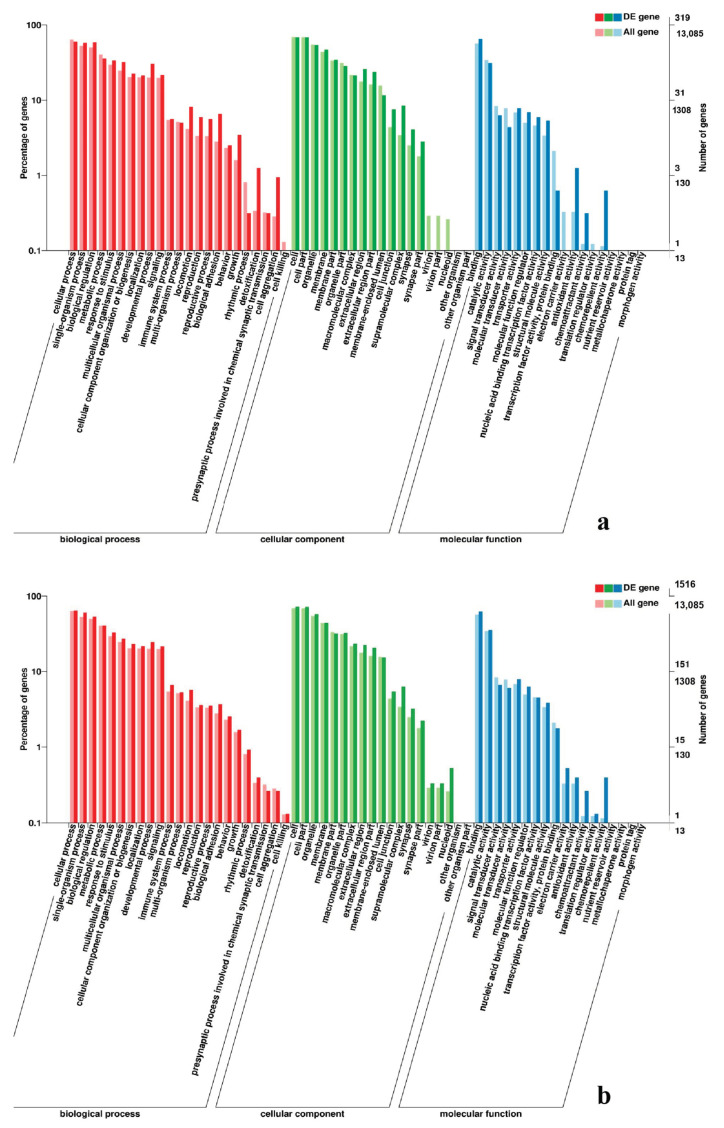
GO enrichment analysis of differentially expressed genes (DEGs) in breast muscle. (**a**) BE17B_vs_BE21B; (**b**) BE17B_vs_BE27B. Note: The abscissa were GO terms, the ordinate on the left was percentage of genes in all genes annotated with GO, right was the number of gene. The same below.

**Figure 4:**
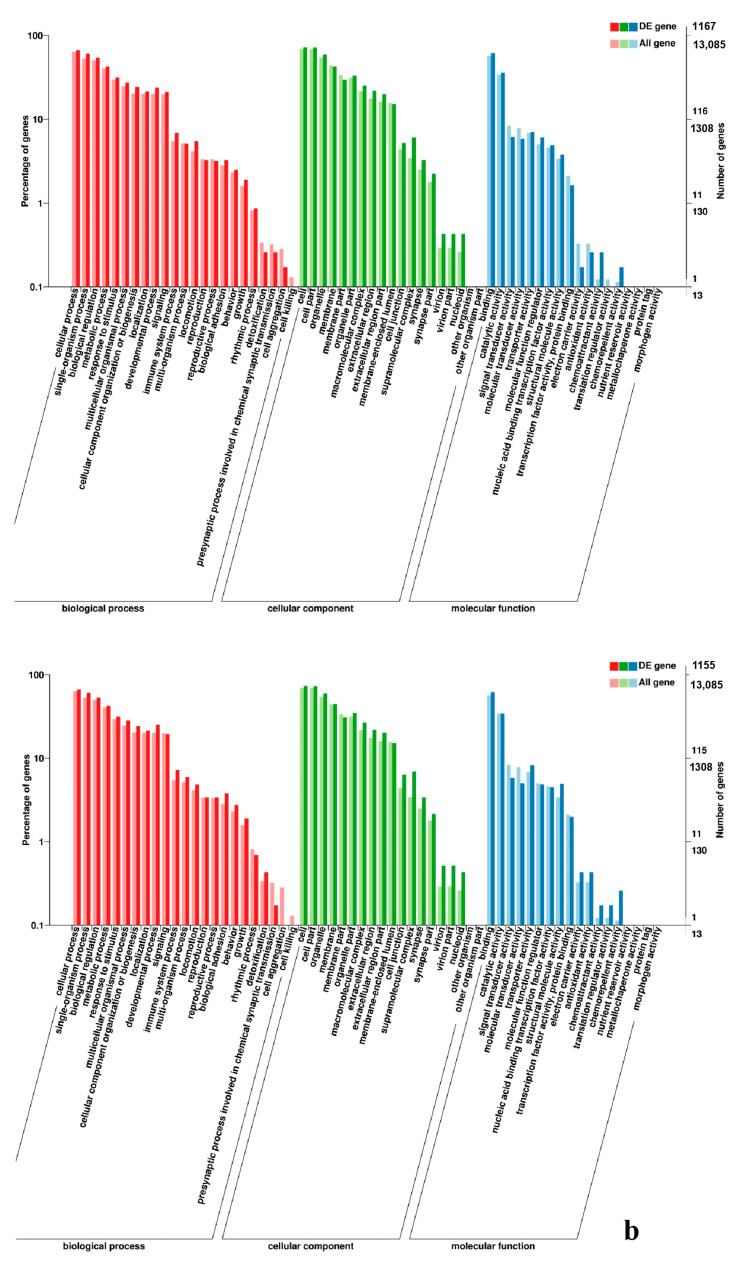
GO enrichment analysis of DEGs in breast muscle (**a**) BE17B_vs_BE31B; (**b**) BE17B_vs_BE34B.

**Figure 5:**
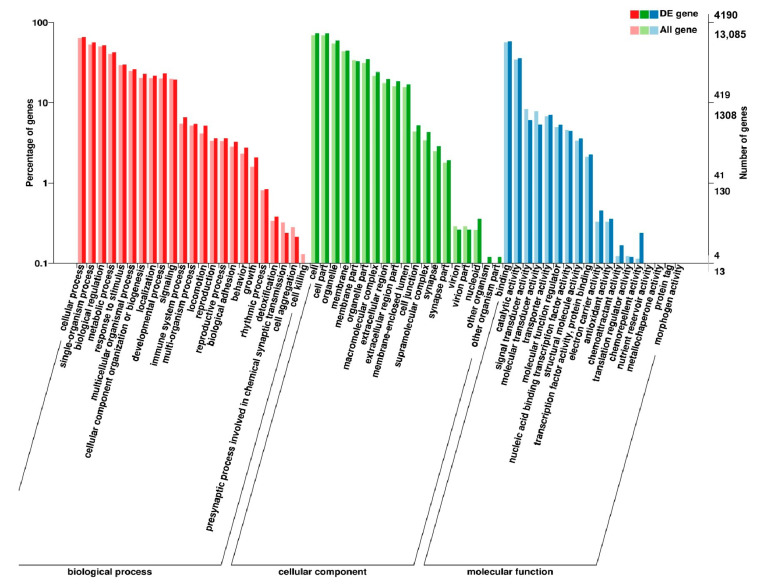
GO enrichment analysis of DEGs in breast muscle of BE17B_vs_BM6B.

To gain valuable insight into the molecular functions of the genes potentially associated with muscle development, the identified DEGs were categorize into three functional groups depending on gene ontology: Biological Process, Molecular Function and Cellular Component. In breast muscle, GO analysis was performed on the common DEGs in BE17B_vs_BE21B indicated that biological processes, including regulation of calcium ion import, regulation of muscle filament sliding speed, dorsal root ganglion development, and negative regulation of fibroblast growth factor receptor signaling pathway were significantly enriched. The common DEGs in BE17B_vs_BE27B were primarily enriched in biological processes of actin binding, positive regulation of myoblast proliferation, cell division, positive regulation of cell proliferation and regulation of actin cytoskeleton organization. Besides, most genes that were involved in the biological processes of regulation of muscle filament sliding, skeletal muscle fiber development, positive regulation of myoblast differentiation and muscle cell cellular homeostasis were mainly enriched in BE31B compared to that of BE17B. The terms of regulation of cell cycle, positive regulation of fibroblast proliferation and positive regulation of substrate-dependent cell migration, cell attachment to substrate were enriched in BE34B compared to BE17B. Additionally, several fundamental biological processes were found to be notably enriched in BE17B_vs_BM6B, such as translation, immune response, regulation of cell size and regulation of cell growth (Figure 3, Figure 4 and Figure 5).

**Figure 6:**
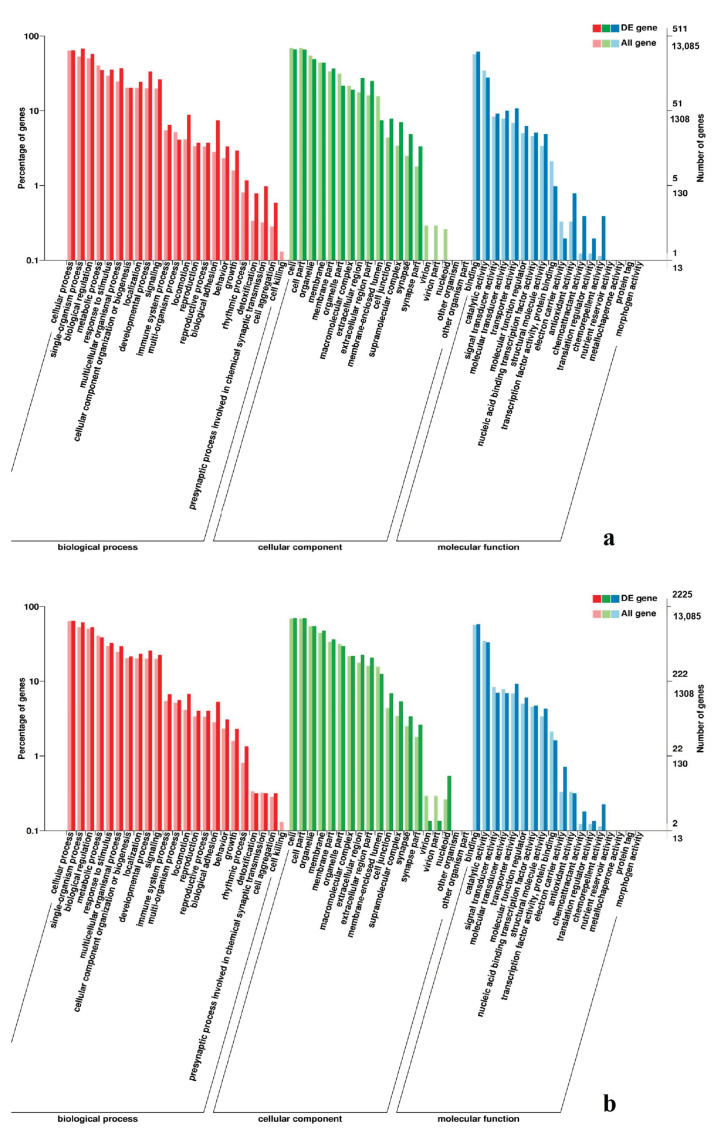
GO enrichment analysis of DEGs in leg muscle. (**a**) BE17L_vs_BE21L; (**b**) BE17L_vs_BE27L.

**Figure 7:**
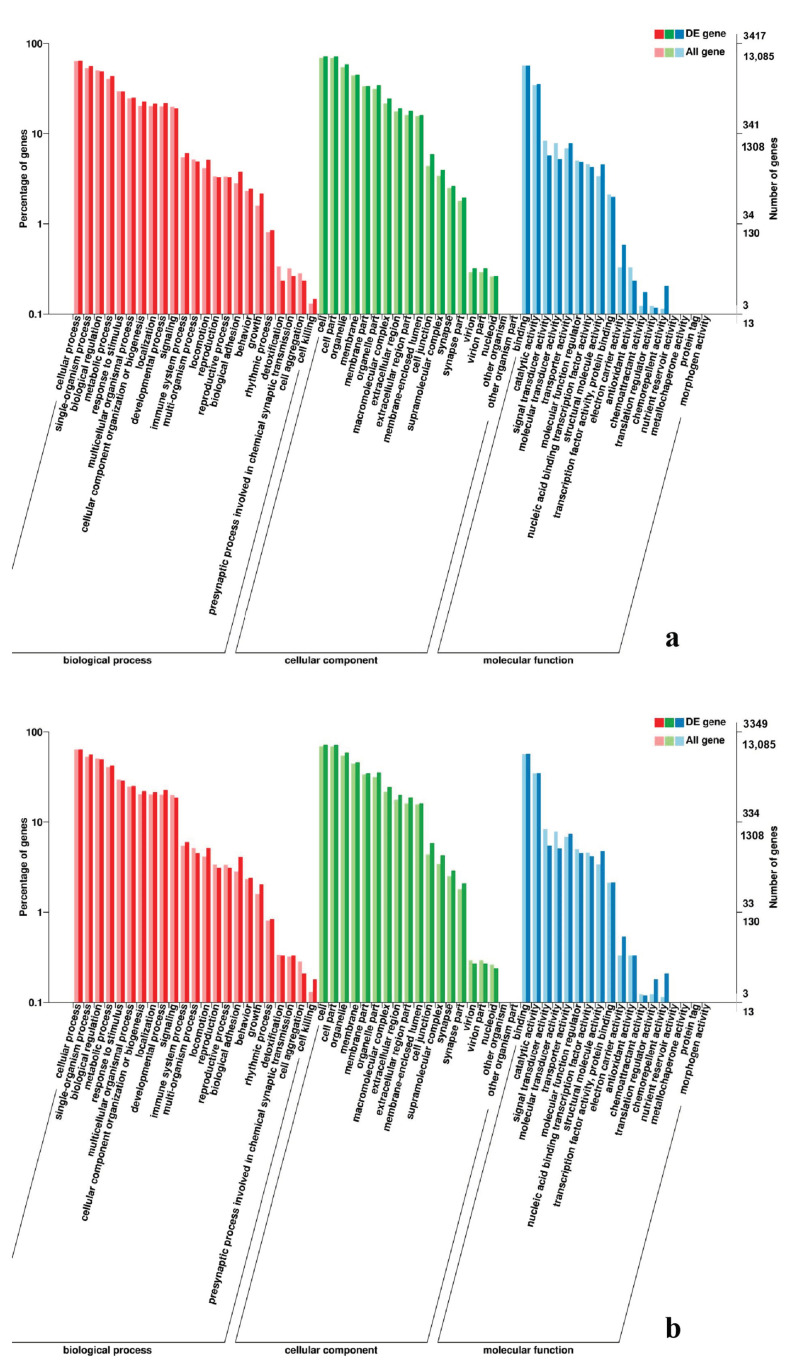
GO enrichment analysis of DEGs in leg muscle. (**a**) BE17L_vs_BE31L; (**b**) BE17L_vs_BE34L.

**Figure 8:**
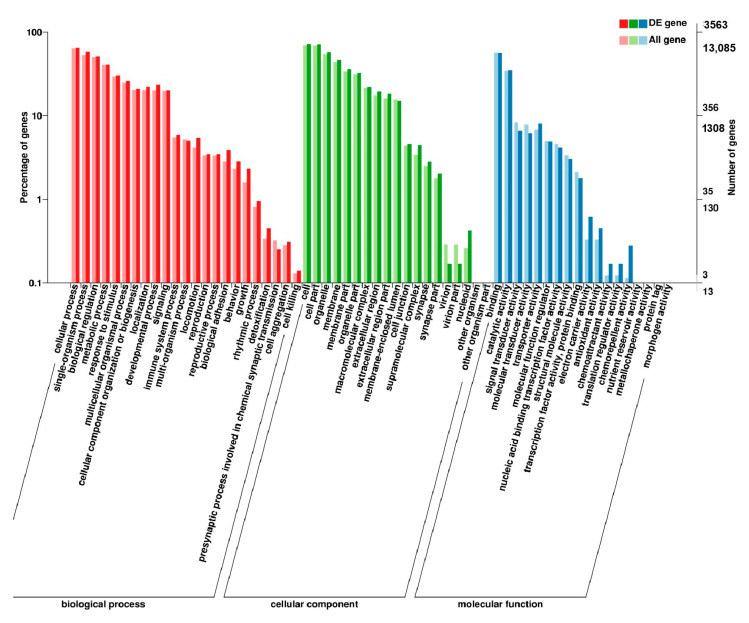
GO enrichment analysis of DEGs in leg muscle of BE17L_vs_BM6L.

**Figure 9:**
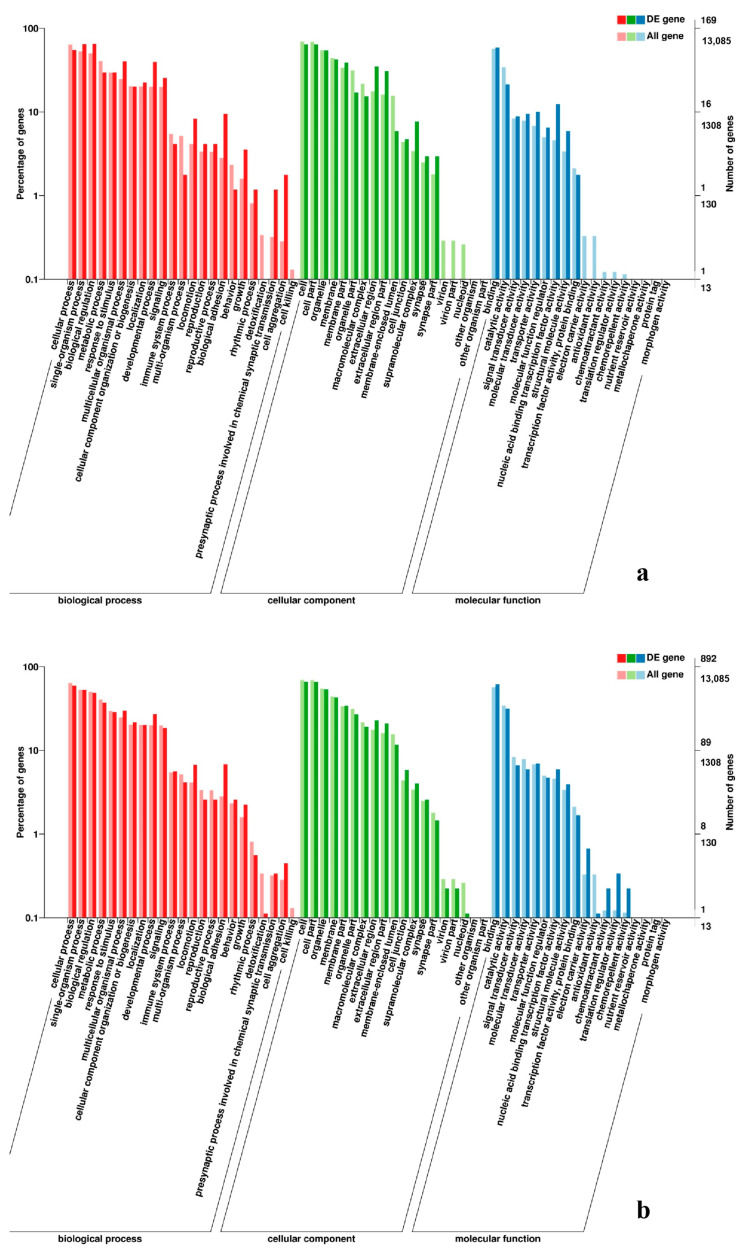
GO enrichment analysis of DEGs in the comparison of breast and leg muscle. (**a**) BE17B_vs_BE17L; (**b**) BE21B_vs_BE21L.

**Figure 10:**
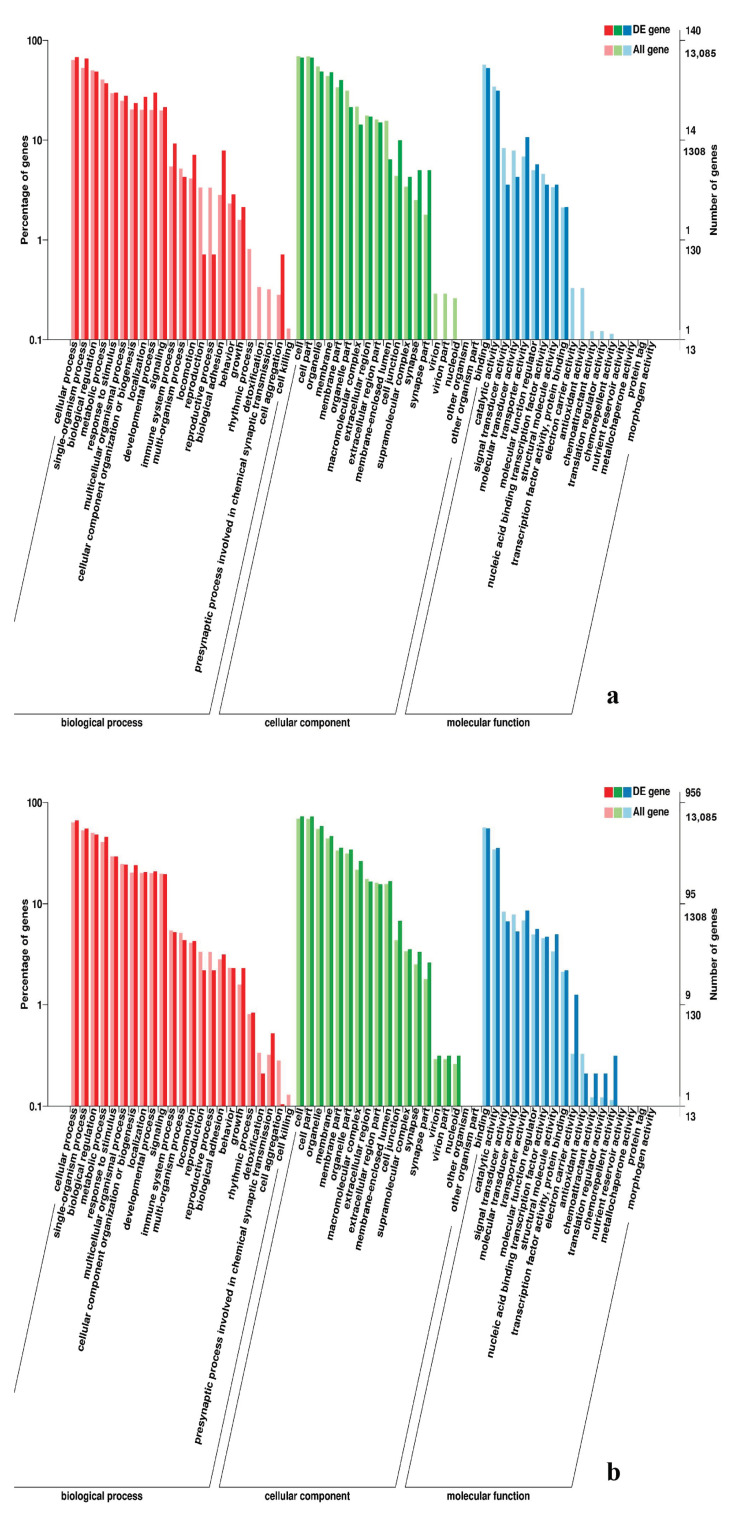
GO enrichment analysis of DEGs in the comparison of breast and leg muscle. (**a**) BE27B_vs_BE27L; (**b**) BE31B_vs_BE31L.

**Figure 11:**
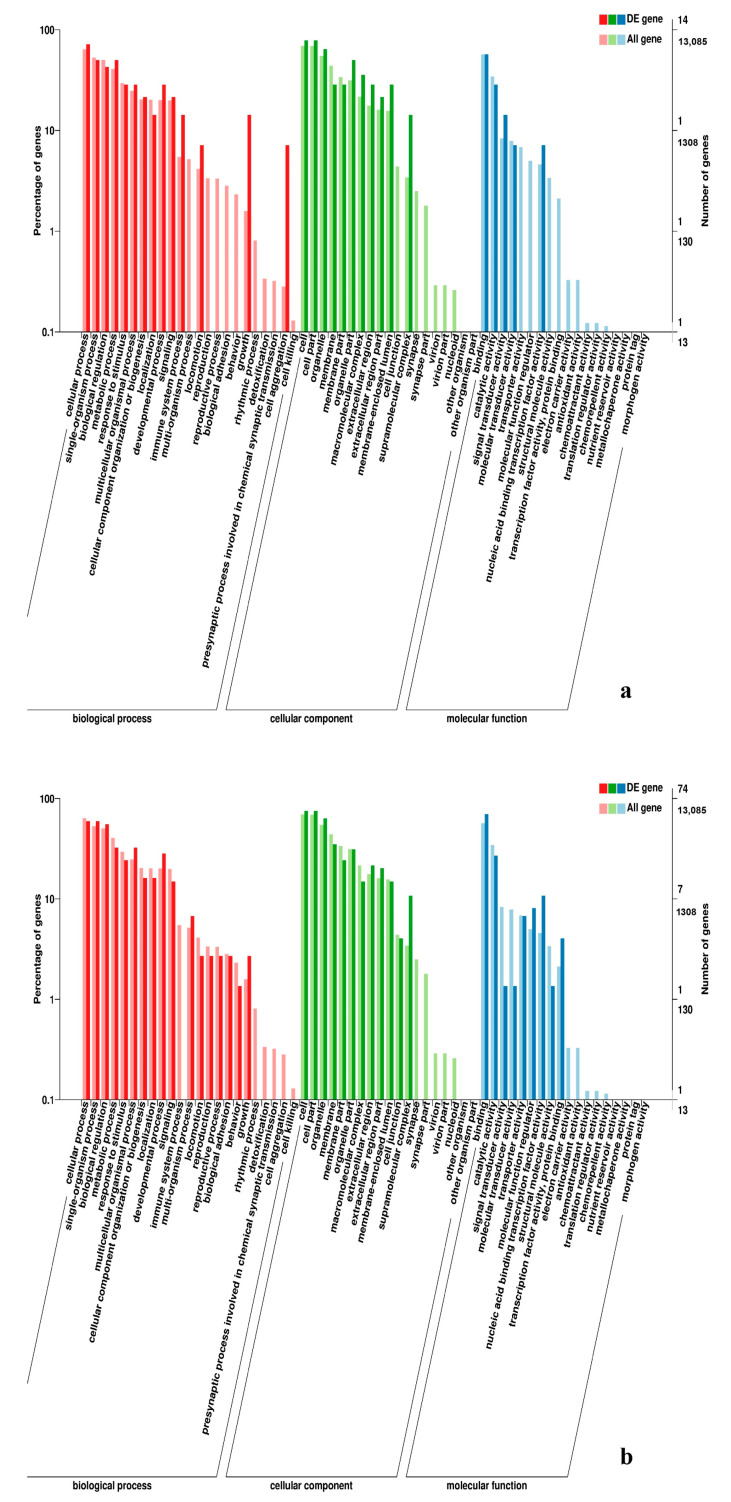
GO enrichment analysis of DEGs in the comparison of breast and leg muscle. (**a**) BE34B_vs_BE34L; (**b**) BM6B_vs_BM6L.

In leg muscle, DEGs were mainly enriched in biological processes of cell proliferation, skeletal muscle fiber development, skeletal muscle tissue development, regulation of muscle filament sliding, positive regulation of cell division in BE17L_vs_BE21L, and regulation of transcription involved in cell fate commitment, calcium-mediated signaling, glucose transport in BE17L_vs_B27L, respectively. Compared to BE17L, most genes that were involved in the biological processes of cell maturation, embryonic limb morphogenesis, chordate embryonic development and immune response were mainly enriched in BE31L. DEGs in BE17L_vs_BE34L and BE17L_vs_BM6L were primarily enriched in biological processes of embryonic hindlimb morphogenesis, positive regulation of protein process, regulation of actin cytoskeleton organization, positive regulation of cell proliferation and Wnt receptor catabolic process, embryonic hindlimb morphogenesis, glucose transport, protein folding, and immune response, respectively (Figure 6, Figure 7 and Figure 8). In the comparison of breast and leg muscle, there were some terms mainly reached in biological processes of myoblast migration involved in skeletal muscle regeneration, positive regulation of glucocorticoid receptor signaling pathways, regulation of multicellular organism growth in BE17B_vs_BE17L, and positive regulation of cellular process, metabolic process, fibroblast migration in BE21B_vs_BE21L, respectively. In BE27B_vs_BE27L and BE31B_vs_B31L, DEGs were primarily enriched in biological processes, including positive regulation of MHC class I biosynthetic process, immune response, metabotic process and regulation of cell shape, embryonic organ development, translation, and muscle structure morphogenesis, respectively. DEGs in BE34B_vs_BE34L were primarily enriched in biological processes of skeletal muscle cell differentiation, skeletal muscle fiber adaptation, myotube differentiation involved in skeletal muscle regeneration, positive regulation of skeletal muscle tissue regeneration. Additionally, several biological processes were found to be notably enriched in BM6B_vs_BM6L, such as muscle structure development, embryonic skeletal joint morphogenesis, myoblast fate commitment, and negative regulation of skeletal muscle tissue development (Figure 9, Figure 10 and Figure 11, Table 6).

**Figure 12:**
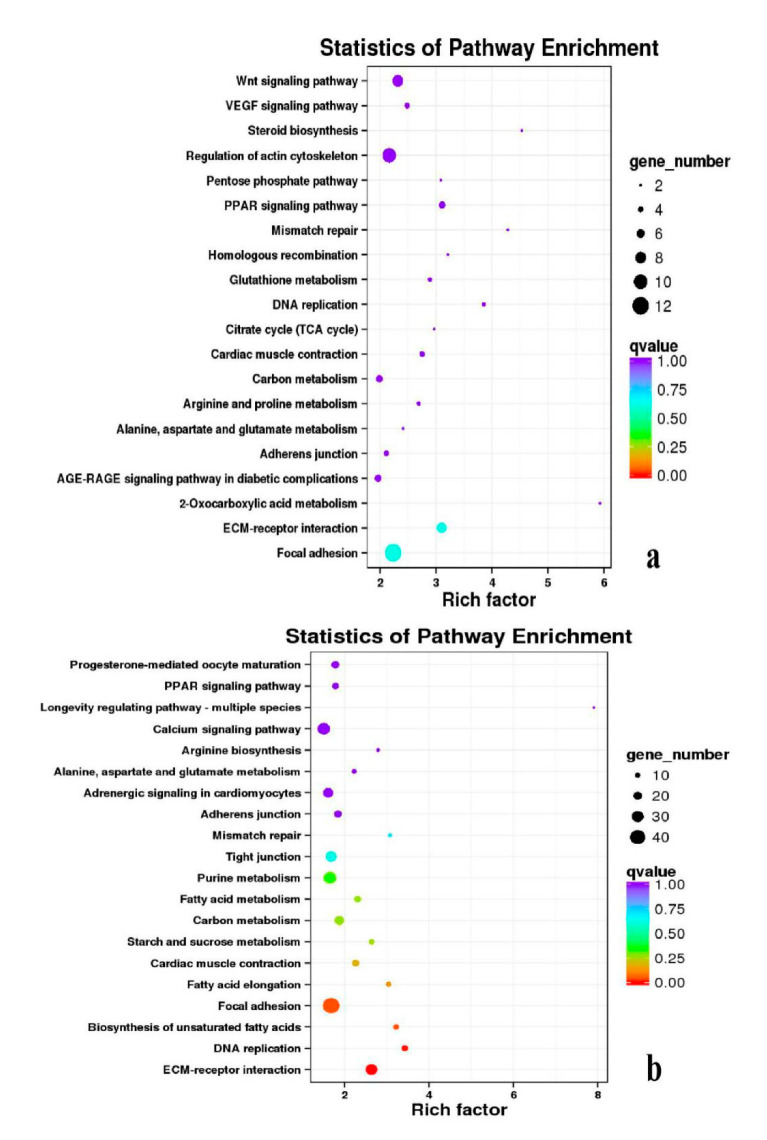
KEGG annotation of DEGs in breast muscle. (**a**) BE17B_vs_BE21B; (**b**) BE17B_vs_BE27B. Note: In the figure, each circle represented a KEGG pathway, name of which was shown on the left legend. Abscissa was enrichment factors, showing the proportion of (**a**) to (**b**), (**a**) was the ration of differentially expressed genes in the pathway with all DEGs in all pathways, (**b**) was the ration of genes in the pathway with all genes in all pathways. The bigger the Rich factor is, the more significant the pathway is. The color of circle represented q value which is adjusted *p* value by multiple hypothesis testing. Thus, the smaller the q value is, the more significant the pathway is; the circle size represented the number of differentially expressed genes annotated with the pathway, the bigger circle size is, the higher number of genes is. The same below.

**Figure 13:**
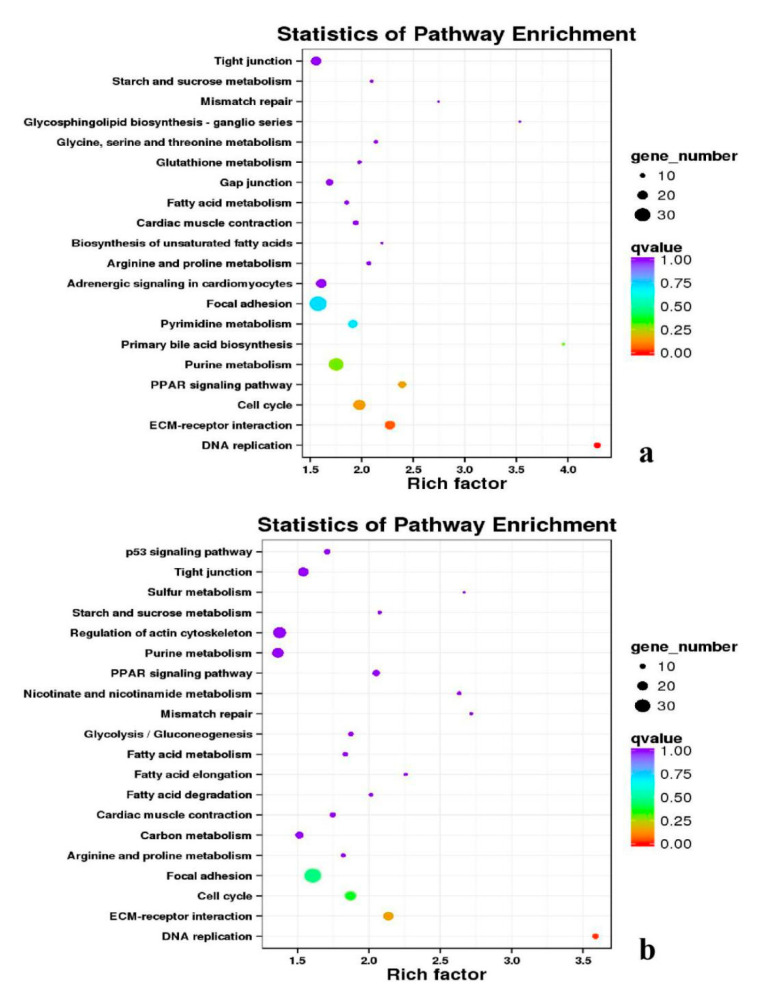
KEGG annotation of DEGs in breast muscle. (**a**) BE17B_vs_BE31B; (**b**) BE17B_vs_BE34B.

**Figure 14:**
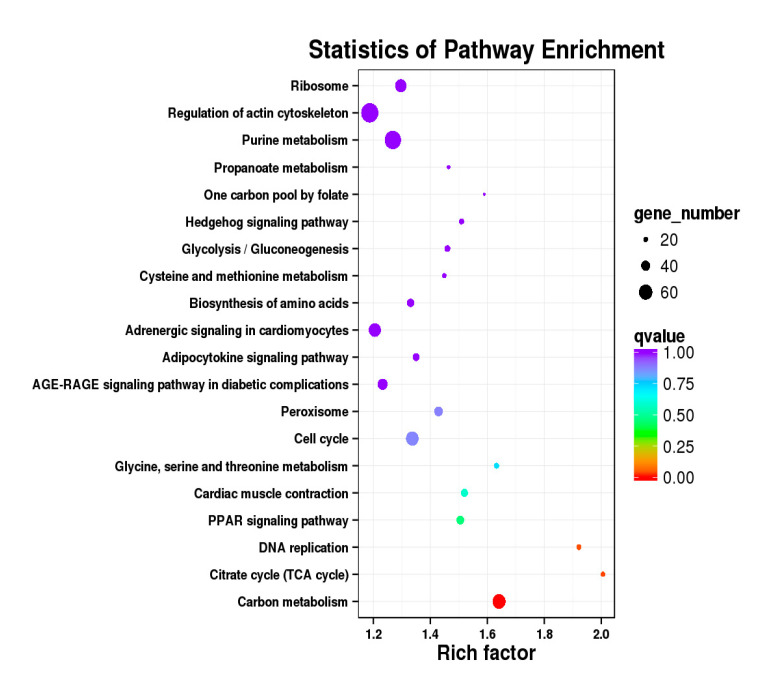
KEGG annotation of DEGs in breast muscle of BE17B_vs_BM6B.

**Figure 15:**
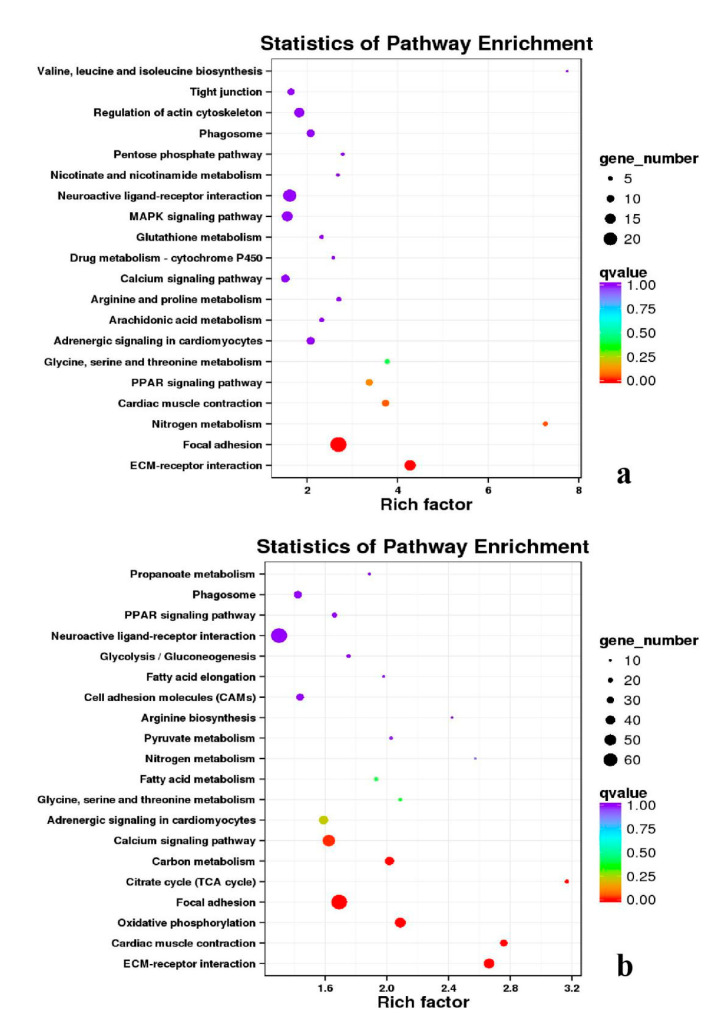
KEGG annotation of DEGs in leg muscle. (**a**) BE17L_vs_BE21L; (**b**) BE17L_vs_BE27L.

**Figure 16:**
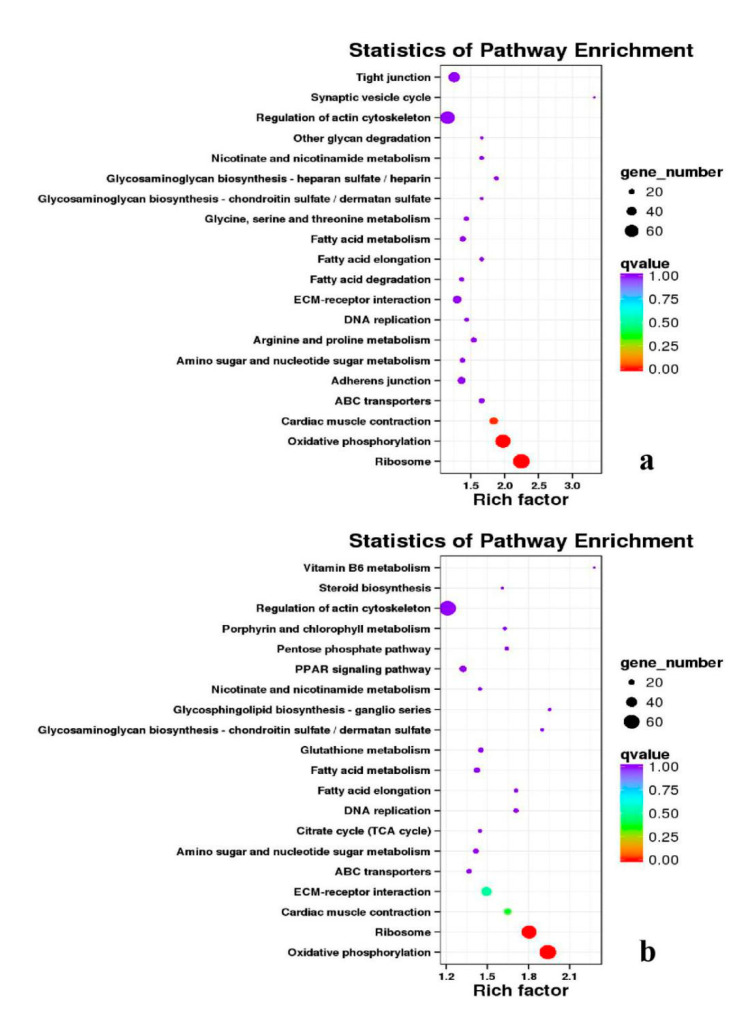
KEGG annotation of DEGs in leg muscle. (**a**) BE17L_vs_BE31L; (**b**) BE17L_vs_BE34L.

**Figure 17:**
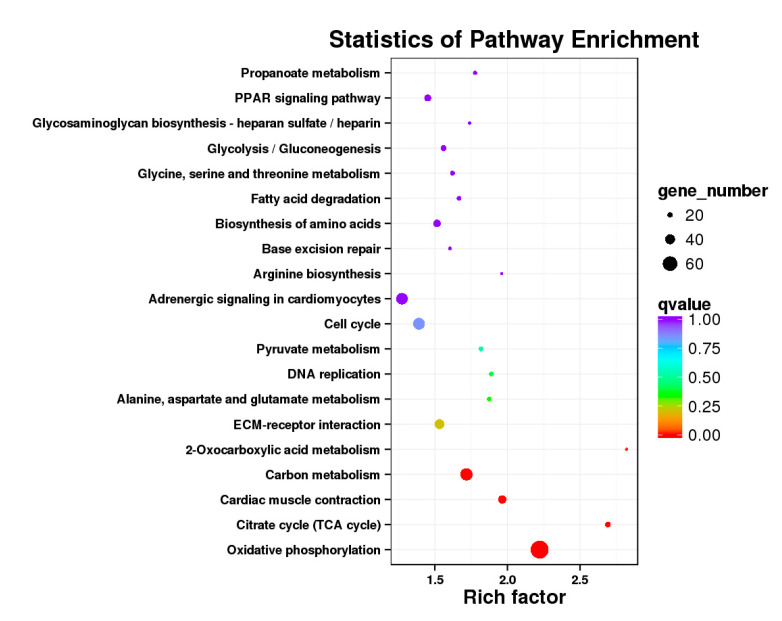
KEGG annotation of DEGs in leg muscle of BE17L_vs_BM6L.

**Figure 18:**
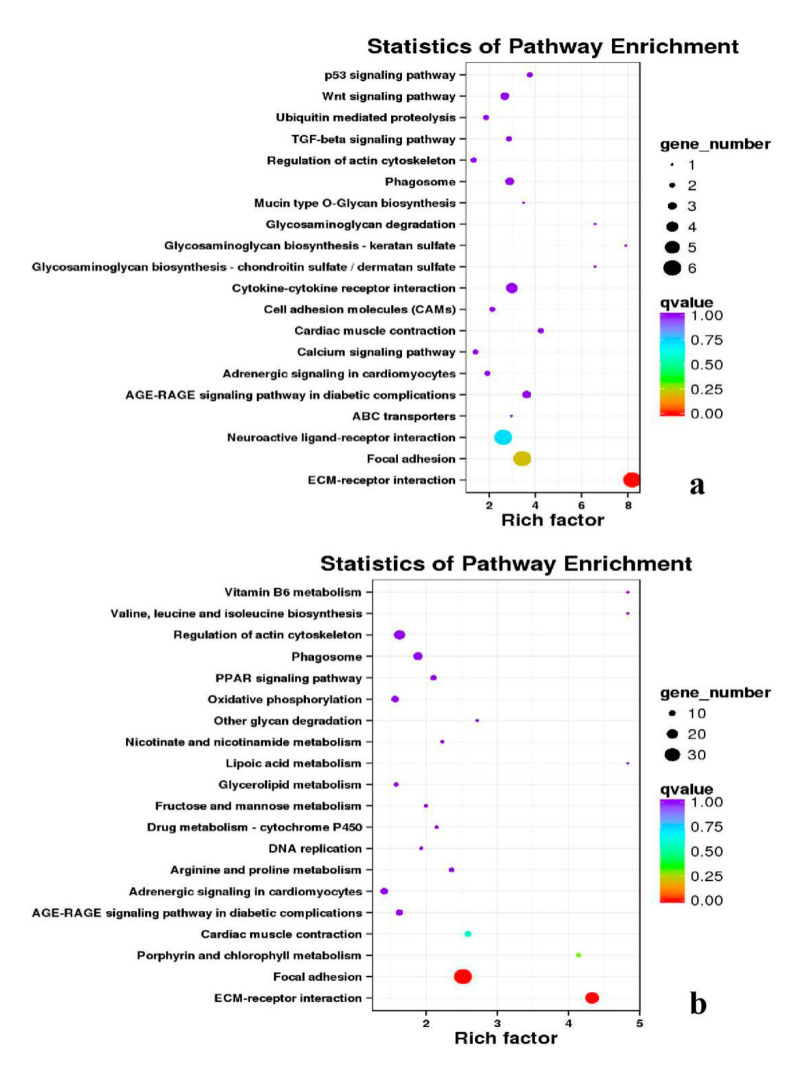
KEGG annotation of DEGs in the comparison of breast and leg muscle. (**a**) BE17B_vs_BE17L; (**b**) BE21B_vs_BE21L.

**Figure 19:**
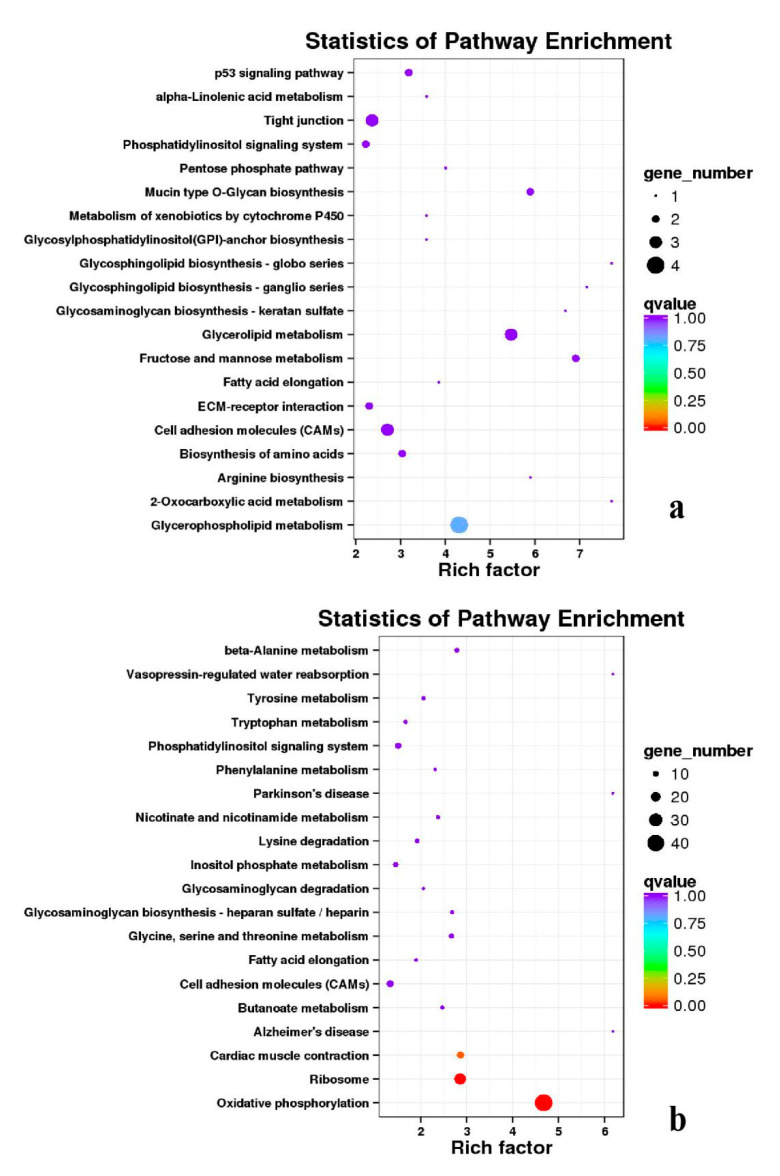
KEGG annotation of DEGs in the comparison of breast and leg muscle. (**a**) BE27B_vs_BE27L; (**b**) BE31B_vs_BE31L.

**Figure 20:**
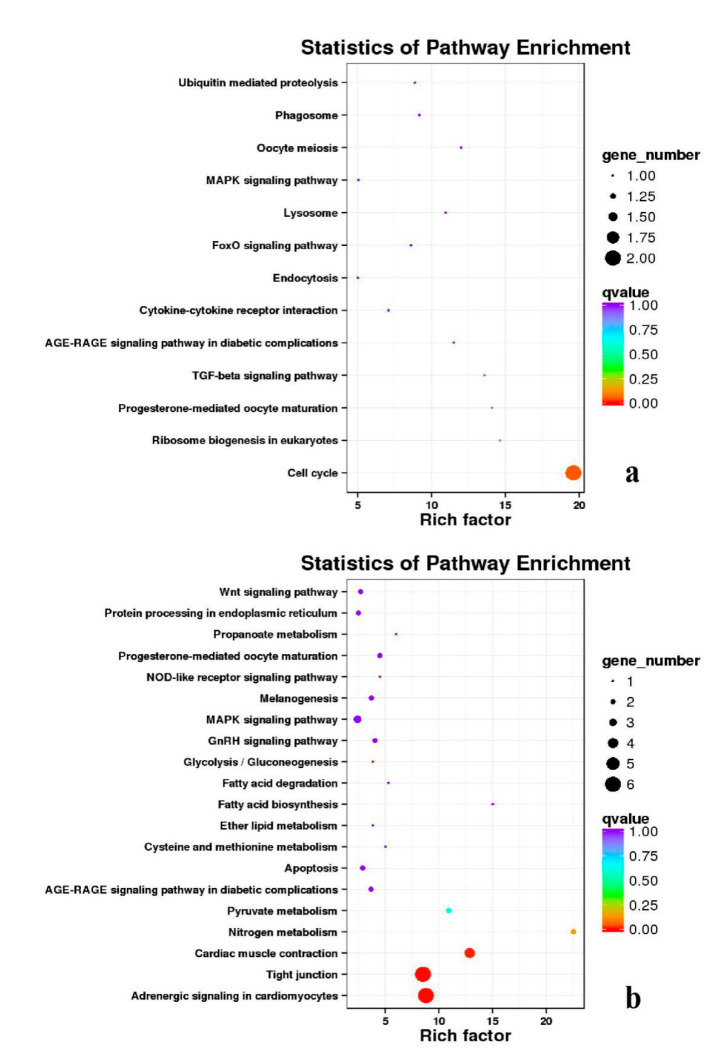
KEGG annotation of DEGs in the comparison of breast and leg muscle. (**a**) BE34B_vs_BE34L; (**b**) BM6B_vs_BM6L.

Besides, Cluster of Orthologous Groups (COG) database was constructed using phylogeny of bacteria, algae, and eukaryotic. The products of genes could be orthologously classified using the COG database (Figures S7–S9). KEGG analysis of DEGs revealed that Focal adhesion, Regulation of actin cytoskeleton, MAPK signaling pathway, Wnt signaling pathway, ECM–receptor interaction, neuroactive ligand–receptor interaction, purine metabolism, calcium signaling pathway, endocytosis, ErbB signaling pathway, glucagon signaling pathway, RIG-I-like receptor signaling pathway, Insulin signaling pathway, cell cycle, apoptosis, oxidative phosphorylation phosphatidylinositol signaling system, cell adhesion molecules (CAMs), biosynthesis of amino acids and Ribosome were the most enriched for the DEGs at different developmental stages (Figure 12, Figure 13, Figure 14, Figure 15, Figure 16, Figure 17, Figure 18, Figure 19 and Figure 20, Table 7).

### 3.6. qPCR Analysis

**Figure 21:**
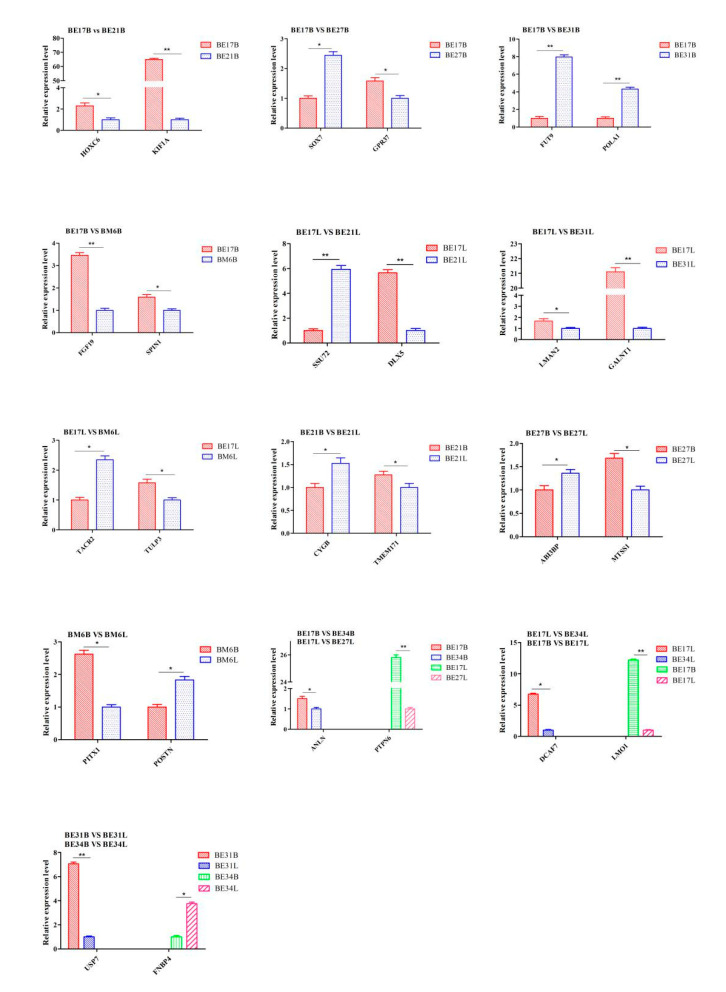
qPCR verification of DEGs. “*” was considered significant difference (*p* < 0.05); “**” was considered extremely significant difference (*p* > 0.01).

To confirm RNA-seq data, 26 genes of duck breast and leg muscle obtained at different growth stages were selected for qPCR analysis, respectively. The expression tendency of these genes agreed well with RNA-seq results, which suggested that RNA-seq results were pretty reliable (Figure 21).

## 4. Discussion

Duck growth performance, mainly skeletal muscle growth, provides direct economic benefits to the poultry industry. However, the underlying genetic mechanisms remain unclear. The purpose of this study was to identify candidate genes associated with the growth of duck skeletal muscle and investigate their potential mechanisms. Transcriptome analysis is an efficient and fast tool that has been widely used in animal husbandry. Comparative transcriptome analyses of tissues at different developmental stages provide valuable insights into the question of how regulatory gene networks control specific biological processes [18,19]. In this study, we focus on the skeletal muscle tissue of black Muscovy duck, because it is the prime part of carcass, and the yield of breast and leg meat is one of practical importance to the profitability of production [20]. Besides, muscle fiber numbers increase during embryonic development in poultry, after which myofiber numbers stop to increase, while myofiber volume still increases [8]. Therefore, we studied the gene expression patterns and network pathways at different developmental stages to further understand the molecular mechanism of duck skeletal muscle development. Annotation of the sequence data using the duck genome as a reference revealed expression of 39,401,532 to 66,211,580 genes in the duck skeletal muscle transcriptome, and an average of 49,119,720 high-quality reads per sample were mapped to the Ap genome.

### 4.1. SNP/InDel Analysis

RNA-seq measures not only gene expression, but also structural variations such as fusion transcriptions or mutations. SNP, a type of genome polymorphisms, only involves the variation of a single base. Because SNPs are abundant and have greater stability over generations, genotyping more accurately and easily automates the genotyping processes [21]. Some studies have reported a large number of SNPs associated with animal weight and muscle development traits. These candidate genes could be used as molecular markers in early marker-assisted selection in animal breeding programs [22,23]. InDels are a major class of genomic variation, which are primarily detected from DNA-seq data [24,25]. In order to explore the gene SNPs and InDels related to muscle growth and development, gene levels of the breast and leg muscle tissues were analyzed by high throughput RNA sequencing technology. There were 23,381 to 634,028 SNPs in skeletal muscles at different growth stages, where total numbers of SNPs in the genic region were 20,572 to 574,484, total numbers of SNPs between genes were 2809 to 59,544. The most common change was G/A and C/T, followed by A/G and T/C. The annotations of SNP were mainly distributed in “INTRON” (2,898,440 for breast muscle and 2,103,619 for leg muscle), “SYNONYMOUS_CODING” (1,319,429 for breast muscle and 1,395,604 for leg muscle) and “UTR_3_PRIME” (1,031,194 for breast muscle and 1,050,568 for leg muscle). The annotations of InDels were mainly distributed in “INTRON” (184,929 for breast muscle and 123,569 for leg muscle), “UTR_3_PRIME” (143,626 for breast muscle and 140,766 for leg muscle) and “DOWNSTREAM” (35,847 for breast muscle and 31,447 for leg muscle), indicating that the skeletal muscle SNPs and InDels of black Muscovy ducks at different growth stages were mainly in “INTRON”, “SYNONYMOUS_CODING”, “UTR_3_PRIME”, and “DOWNSTREAM”. Inclusion of SNPs and InDels (many of which may be located in genes) in annotated genes will provide a large number of gene centric markers, which will add detailed information for the genetic loci for specific phenotypic traits.

### 4.2. Prediction of AS

AS is a ubiquitous in most eukaryotic genomes, which is a mechanism for organisms to increase their protein pool and regulate physiological and developmental processes/pathways [26,27,28]. Precursor mRNA of AS plays an important role in the regulation of gene expression in higher eukaryotes. Multiple mRNAs can be derived from a single pre mRNA to produce proteins with different functions, which indicates that AS is an important mechanism for regulating life [29]. Isoform expression patterns may provide unique insights into the skeletal muscle transcriptome since it is likely that unique transcript splice variants may play essential roles during development [30]. Due to the lack of detailed full-length cDNA data and high-quality genome annotation, there are few studies about AS in ducks or other birds [31,32]. Yin et al. (2019) found that a total of 199,993 full-length transcripts were obtained from 8 duck tissues by using transcriptome sequencing, and 35,031 AS events were accurately predicted from 3346 genes, which is very useful for the functional research of other birds [33]. In our study, an average of 37,267 AS events were counted from each sample, and the number of AS in each sample were mainly concentrated in TSS and TTS, which indicated that the AS of duck skeletal muscle growth and development mainly existed in the first exon and the last exon.

### 4.3. DEGs Analyzed at All Time Points

The differential expression of growth related genes is considered to be the main cause of genetic variation during duck growth and development, indicating that the regulatory mechanism of growth has changed [13]. In our study, at different time points, the skeletal muscle of black Muscovy duck had 19 to 5377 DEGs. It was worth noting that there were only 19 DEGs in BE34B_vs_BE34L. It is speculated that the genes that regulate the proliferation and differentiation of duck breast and leg muscle were basically the same at this time. It is well known that these processes involved by the DEGs at different developmental stages are essential for maintaining animal muscle growth. Therefore, these genes may play an important role in growth and development of duck skeletal muscle. Further functional studies with these DEGs were warranted to identify key genes influencing muscle growth and development of duck. Besides, some DEGs were found to be closely related to growth and development of skeletal muscle in black Muscovy duck, including transcription factors such as *MyoG*, *FBXO*1, *MEF2A*, and *FoxN*2, as well as a series of genes related to muscle growth axis, such as *FAF*1, *RGS*8, *GRB*10, *SMYD*3, and *TNNI*2. Moreover, some studies have shown that these regulatory transcription factors interacted with each other in regulating animal muscle growth.

Fas-associated factor 1 (FAF1) is involved in diverse bio-chemical processes including cell death, inflammation, cell proliferation, and proteostasis [34]. FAF1 mediates caspase-8 activation via both intrinsic and extrinsic pathways. It suppresses NF-κB activation by interrupting IκB kinase (IKK) complex assembly, and promotes Fas-induced apoptosis [35,36]. *FAF*1 also inhibits cell cycle by negatively regulating Aurora-A. Moreover, FAF1 regulates the polyubiquitinated protein and valosin containing protein, and inhibits the degradation of ubiquitin dependent proteins [37,38]. Studies have shown that *FAF*1 antagonized Wnt signal transduction by promoting the degradation of β-Catenin [39]. *Wnts* regulate cell proliferation and differentiation, and control many biological processes including embryonic development [40,41]. *FAF*1 regulates Wnt/β-Catenin dependent gene expression in C2C12 myoblasts, including genes involved in osteoblast differentiation [39]. *FAF*1 is necessary for early embryogenesis. Adham et al. (2008) found that *FAF*1 gene targeted mice showed embryonic lethality at the two cell stage [42]. Ryu et al. (1999) found Human *FAF*1 mRNA is abundantly expressed in testis, skeletal muscle, and heart [43]. Fröhlich et al. (1998) identified the avian *FAF*1 homologue (qFAF) in the pluripotent cells from E0 quail embryos during mesoderm induction in vitro by using mRNA differential display technique, which can be used as the induction gene of fibroblast growth factor (*FGF*) [44]. Therefore, *FAF*1 may play an important role in the development of duck skeletal muscle.

Regulator of G protein signaling proteins (RGS) are negative modulators of many G-Protein Coupled Receptor (GPCR) signaling pathways [45]. RGS protein directly binds to the Gα subunit of GTP of activated heterotrimer G protein, which increases the rate of GTP hydrolysis [46]. By this mechanism, RGS proteins rapidly dampen GPCR signal transduction at the level of the active G protein subunits [47]. Regulator of G protein signaling 8 (RGS8) belongs to the R4 subfamily of RGS proteins, and modulates the functioning of G-proteins by activating the intrinsic GTPase activity of the a subunits [48,49]. *RGS* is involved in many intracellular processes mediated by G protein signal transduction pathway, including cell proliferation, cell differentiation, plasma membrane transport, cell movement, and embryonic development [50]. In our study, *RGS*8 may regulate G protein by activating GTPase activity of a subunit and participate in development of duck skeletal muscle.

Growth factor receptor-bound protein 10 (GRB10) regulates phosphorylation and activation of the mTORC1 protein, which is a central regulator of cellular metabolism, growth and survival [51,52]. GRB10 is also involved in the regulation of glucose metabolism during fetal and postnatal period [53]. It is also one of the participants in ensuring the metabolic health of fetus to adult. *GRB*10 binds to *Gab*1 and participates in the regulation of cell mitosis [54]. Besides, GRB10 is an adaptor protein, which interacts with a number of receptor tyrosine kinases and signaling molecules [55]. GRB10 associates with a variety of growth factors at the cell surface, such as the insulin growth factor receptor (IGFR), and with intracellular protein kinases like Raf1 and MEK1 [56,57]. It is a negative regulator of IGFIR-dependent cell proliferation, and plays a negative regulatory role in the MAPK signaling pathway [58,59]. Notably, IGF and MAPK signaling pathway play an important role in skeletal muscle development. Studies have shown that *GRB*10 is abundant in brain, fat, muscle and heart of mice [60]. Mutation of *GRB*10 induces muscle hypertrophy in mice [61], and the embryos and placentas of mice lacking *GRB*10 were overgrown, such that mutant mice were 30% larger than normal at birth [55]. It was suggested that *GRB*10 may be very important for skeletal muscle development in duck.

Muscle fibers are composed of myofibrils, and members of the SMYD family play critical roles in myofibril assembly of skeletal and cardiac muscle during development [62]. SET and MYND domain-containing protein 3 (SMYD3) is widely distributed in eukaryotes and participates in epigenetic transcription regulation, development and cell proliferation [63]. *SMYD*3 is also necessary for regulating skeletal muscle and myocardial development [64]. *SMYD*3 controls skeletal muscle development and maintenance through transcriptional regulation [65]. Fujii et al. (2011) found that in zebrafish embryos, *SMYD*3-knockdown led to abnormal expression of myogenic markers including *MyoD*, which indicated that *SMYD*3 may play a role in muscle development [64]. In addition, *SMYD*3 is involved in regulating skeletal muscle atrophy. During the process of dexamethasone-induced skeletal muscle atrophy, the mRNA level of *SMYD*3 was significantly increased [66]. The differential expression of *SMYD*3 in duck may be related to greater myogenic potential. Troponin I2 (*TNNI*2), a muscle growth marker gene [67,68], encodes a subunit of troponin complex [69], which are expressed under muscle type-specific and developmental regulations [70]. Troponin complex is a group of muscle proteins, which is part of the contraction device of rapid skeletal muscle contraction [71]. In the absence of Ca^2+^, *TNNI*2 prevents muscle contraction by binding actin and tropomyosin [72]. Besides, Yoshimoto et al. (2020) found that expression of *TNNI*2 was gradually increased as muscle regeneration proceeds, indicating that *TNNI*2 were excellent indicator to assess myofiber maturity [73]. *TNNI*2 encodes an isoform of *TnI* specific to fast-twitch myofibers and troponin I (*TnI*) mutation with abnormal skeletal muscle structure leads to wing and limb defects in *Drosophila* [74]. Besides, the expression of slow-twitch *TnI* is stronger in soleus muscle, while the expression of fast-twitch *TnI* is stronger in tibial anterior muscle and extensor digitorum longus in neonatal mice [75]. Therefore, *TNNI*2 may be an interesting candidate gene to explain the phenotypic differences of skeletal muscle development in duck.

The RNA-Seq data were confirmed to be reliable by qPCR. These identified DEGs included many genes significantly related to muscle development.

### 4.4. GO and KEGG Pathway

GO database is a structured standard biological annotation system. It aims to establish a standard system for genes and their products, which is suitable for all species. GO annotation system includes three main branches: Biological Process, Molecular Function, and Cellular Component. In this study, several key DEGs (such as *MyoG*, *FBXO1*, *MEF2A, FAF1*, *RGS8*, *GRB10*, *SMYD3*, and *TNNI2*) involved in muscle development can be identified in enriched GO terms, which providing a theoretical basis for the skeletal muscle development of black Muscovy duck at different growth stages. The GO analysis of these DEGs showed that the biological processes related to cell growth, muscle development, and cellular activities (such as junction, migration, assembly, differentiation, and proliferation), muscle contraction, as well as glycogen metabolic and biosynthetic processes, were regulated differently at each developmental stage, indicating that the DEGs played an important role in regulating duck skeletal muscle.

In organisms, different genes interact to perform biological functions. Annotation and analysis of pathways are helpful to further understand the functions of genes. KEGG is a database for systematic analysis of gene function and genome information, which can be used as a whole network to study gene and expression information. Duck muscle growth is a complex process influenced by multiple genes and controlled by multiple pathways. In our KEGG analysis, main pathways related to muscle growth were identified, namely, focal adhesion, ECM–receptor interaction, MAPK signaling pathway, neuroactive ligand–receptor interaction, endocytosis, oxidative phosphorylation, ribosome, tight junction, insulin signaling pathway, and regulation of the actin cytoskeleton, of which the focal adhesion, MAPK signaling pathway and regulation of the actin cytoskeleton were the most significantly enriched (Figures S10–S12).

Focal adhesions (FAs) are integrin-containing, multi-protein assemblies crossing the plasma membrane that connect the cellular cytoskeleton to surrounding extracellular matrix. They play a key roles in adhesion and cell signal transduction, and are the main regulators of epithelial homeostasis, tissue response to injury and tumorigenesis [76]. Focal adhesion kinase (FAK) is a multifunctional molecule with the ability to regulate muscle formation, hypertrophy and glucose metabolism [77]. The mitogen-activated protein kinase (MAPK) signaling pathway is a phosphorylation kinase signaling cascade that regulates many cell processes, such as cell division, differentiation, and release of inflammatory mediators [78]. It is well known that MAPK signaling pathway induces protein synthesis and promotes skeletal muscle growth or hypertrophy [79]. The actin cytoskeleton comprises a scaffold of polymeric actin filaments that are assembled and disassembled to organize cell architecture and direct many cell processes. The actin-related proteins control actin filaments reorganization, resulting in significant changes in actin cytoskeleton structure, thus regulating cell processes that affect mitosis, cytokinesis, endocytosis, and cell migration [80,81]. At focal adhesion, the extracellular domain of transmembrane integrin, including α and β subunits, is connected to the extracellular matrix (ECM). The intracellular tail of integrin binds to connexin, and connexin binds to the actin cytoskeleton to perform their related functions [82]. Therefore, the results of this study will allow us to predict the function of new genes and to explore candidate genes that might play a role in muscle growth and development process in duck. Besides, the annotation findings of the current investigation can be used as the selection marker for the genes related to the skeletal muscle growth and might be helpful in better understanding of genetic mechanisms associated with the growth of duck skeletal muscle.

## 5. Conclusions

In this study, transcriptome data was generated by RNA-Seq technology, which will help to further understand the molecular sequences and functions of skeletal muscle growth related genes of black Muscovy duck at different stages. There were differences in the expression of genes at different growth stages, including SNPs, InDels, AS, highly expressed genes and pathways. These findings will provide valuable resources for the biological researches of skeletal muscle growth related genes in black Muscovy duck and may also provide clues for understanding the molecular mechanisms in other poultry and mammalian species.

## Figures and Tables

**Table 1:** qPCR primer sequences of black Muscovy duck.

Groups	Primer Name	Primer Sequence (5′-3′)	Amplicon Size	Regulated
	gCHD	F: TGCAGAAGCAATATTACAAGT	Male: 467 bp Female: 467 bp, 326 bp	
		R: AATTCATTATCATCTGGTGG		
BE17B_vs_BE21B	*HOXC*6	F:CCAAAACAGGAACACTTCGCA	167 bp	Down
		R:AAAAGTCGCTCAGCCTGTTCT		
	*KIF1A*	F:AAAGGGCTACCTGCACTTCC	188 bp	Down
		R:CTGCACCCACCTTCAGCAT		
BE17B_vs_BE27B	*SOX*7	F:AGATGGACCGCAACGAAT	150 bp	Up
		R:CAGCAAGGACGGAGATGA		
	*GPR*37	F:CGCCAGTCCTCCTTTTCTGT	175 bp	Down
		R:ATTTCACGACGGATGGTGCT		
BE17B_vs_BE31B	*FUT*9	F:GACGTACTTGGTCTGGGTCA	158 bp	Up
		R:GCACCCCACCTTACAACCTC		
	*POLA*1	F:CCGCTCAGAAAGGAGGTGATT	172 bp	Up
		R:CTCCCTTTTCAGCCCATCACT		
BE17B_vs_BE34B	*ANLN*	F:TTCCAGGACAAGGTTCCTGTT R:AGTTTATCCGGCCCAAAGGAT	229 bp	Down
BE17B_vs_BM6B	*FGF*19	F: TGTCTTTGCTTGGCGCTACT R:CAGTGTACGGTGTGGTTGAGT	214 bp	Down
	*SPIN*1	F:TCGGATTAGTGATGCCCACC R:CTGGCCTACTTACTGGAATCGG	240 bp	Down
BE17L_vs_BE21L	*SSU*72	F:CAAGCCACGACCAGAGAGAT R:GGGTTGCCTCCTCATGGTTA	176 bp	Up
	*DLX*5	F:ACCCTGCTGTGCGTAAGA R:GGAAAGGAGCCTGGAAGT	232 bp	Down
BE17L_vs_BE27L	*PTPN*6	F:TCTCCTATCCCGTGAGCCAA R:ATTTTCTGCCCACCCCTAGC	131 bp	Down
BE17L_vs_BE31L	*LMAN*2	F:GGGAGTTTTCCTTGCCCCAG R:GTTGGTTCACTTTGTTCTGCCC	196 bp	Down
	*GALNT*1	F:AGGGGAAGGTCGGGAAAGTT R:ACAGGCAGTCCTCCTACTCAA	201 bp	Down
BE17L_vs_BE34L	*DCAF*7	F:GTACAGCAGGTAGGTGTGGAA R:TGCCATCCAATAAGCAGGCAT	226 bp	Down
BE17L_vs_BM6L	*TACR*2	F:CATCGCAGTGATCGTGTTGA R:CGTGCAAGCTCTGTGTTGGA	229 bp	Up
	*TULP*3	F:GGCCACTGGTAATGACATGCT R:GTAGCTCGCTCCAAAGACAGT	109 bp	Down
BE17B_vs_BE17L	*LMO*1	F:GCGATTCTGTGTGGGAGACA R:TTGAACCTGGGACTCGAAGC	106 bp	Up
BE21B_vs_BE21L	*CYGB*	F:GAGGCGGAGAAGAAGGTGATT R:CGTGTCGTCCATGTGCTTGA	147 bp	Up
	*TMEM*171	F:CTGATGTGAACCTCCAGGGC R:TGGTGGTGGAGGTGGGAATA	218 bp	Down
BE27B_vs_BE27L	*ABI3BP*	F:CGAAACCATCTGCTACCCCA R:TGACTGACACCGGAATGGC	213 bp	Up
	*MTSS*1	F:TACAGCACCCAGACGACAAC R:AAACTCTTGCTGCTCTGCCT	114 bp	Down
BE31B_vs_BE31L	*USP*7	F:GTCTGTCCGGGTAGAGTCGT R:GAATACACACCCATGTTGCAGG	242 bp	Down
BE34B_vs_BE34L	*FNBP*4	F:ACGAAAATGCCGTCTCTGGT R:CGAAGTTGGCGTTCCTCTCT	172 bp	Up
BM6B_vs_BM6L	*POSTN*	F:GCAGGGAGCTGGAACTGAG R:TGTTGCTCCTCCTTGTGTCC	148 bp	Up
	*PITX*1	F:AGCACTCCAGTTTCGGCTAC R:CTCACTTGCTCGGGTTTTGC	226 bp	Down
	*β-actin*	F: CCCTGTATGCCTCTGGTCG R: CTCGGCTGTGGTGGTGAAG	194 bp	

Note: BE17B: Breast muscle of black Muscovy duck on day 17 of the incubation period; BE17L: Leg muscle of black Muscovy duck on day 17 of the incubation period. The same below.

**Table 2:** The feed composition of black Muscovy duck.

Ingredient	Content (%)	Nutrient	Content (%)
Corn	56.00	Crude protein	15.700
Soybean meal	23.80	Calcium	0.900
Corn gluten meal	10.00	Total phosphorus	0.680
Limestone	7.00	Available phosphorus	0.450
CaHPO_4_	1.50	Salt	0.370
Premix	1.00	Lysine	0.760
NaCl	0.30	Methionine	0.387
Lys·HCl	0.30	Methionine + Cystine	0.654
*DL*-Met	0.10	Isoleucine	0.534
Total	100.00	Threonine	0.579
		Tryptophan	0.194
		Crude fiber	4.100
		Crude fat	3.400
		Crude ash	5.200
		Avian metabolizable energy	2875 Mcal·kg^−1^

**Table 3:** RNA-Seq data from breast muscle and leg muscle of black Muscovy duck.

Samples	Clean Reads	Clean Bases	GC Content	≥Q30 (%)
BE17B1	26,764,472	7,980,103,934	50.51%	93.57%
BE17B2	32,024,936	9,563,668,162	50.63%	93.35%
BE17B3	23,594,320	7,046,658,352	50.99%	92.86%
BE17L1	26,550,573	7,917,527,738	50.81%	93.07%
BE17L2	33,105,790	9,881,964,696	51.01%	92.80%
BE17L3	22,233,811	6,608,083,230	50.44%	93.75%
BE21B1	21,356,816	6,372,442,332	50.93%	93.10%
BE21B2	23,023,064	6,874,209,792	50.50%	92.30%
BE21B3	22,232,157	6,643,515,660	50.10%	91.93%
BE21L1	27,233,943	8,132,501,326	50.35%	92.25%
BE21L2	23,821,161	7,114,195,066	50.77%	92.71%
BE21L3	25,252,424	7,545,013,174	51.35%	92.99%
BE27B1	26,005,019	7,766,530,628	51.05%	92.74%
BE27B2	26,902,913	8,034,045,734	50.82%	92.73%
BE27B3	24,226,391	7,227,540,800	51.53%	92.75%
BE27L1	23,634,707	7,060,587,498	51.47%	92.59%
BE27L2	25,561,499	7,630,071,354	51.34%	93.31%
BE27L3	29,341,501	8,760,492,104	51.16%	93.13%
BE31B1	21,708,384	6,481,320,616	50.43%	93.32%
BE31B2	21,045,091	6,284,463,090	50.48%	92.65%
BE31B3	23,666,137	7,071,687,724	50.01%	93.80%
BE31L1	21,773,785	6,504,542,434	50.79%	92.41%
BE31L2	19,700,766	5,883,221,398	50.94%	92.35%
BE31L3	25,067,398	7,480,770,550	50.95%	92.68%
BE34B1	19,970,569	5,959,632,696	51.20%	91.89%
BE34B2	21,641,494	6,468,354,774	50.57%	92.51%
BE34B3	24,247,593	7,240,167,310	50.04%	91.36%
BE34L1	19,825,346	5,925,722,250	49.19%	92.01%
BE34L2	20,622,571	6,154,061,186	50.82%	91.72%
BE34L3	21,504,291	6,418,738,234	50.92%	91.70%
BM6B1	27,888,927	8,312,322,988	52.05%	93.09%
BM6B2	26,315,704	7,841,240,550	51.31%	93.29%
BM6B3	32,141,926	9,592,590,862	52.51%	92.91%
BM6L1	22,567,829	6,744,703,784	56.80%	92.26%
BM6L2	25,330,860	7,561,056,178	53.01%	92.95%
BM6L3	26,270,786	7,826,518,320	50.75%	93.01%

**Table 4:** Single nucleotide polymorphism (SNP) statistics of each sample.

Samples	SNP Number	Genic SNP	Intergenic SNP	Transition	Transversion	Heterozygosity
BE17B1	483,071	442,220	40,851	71.88%	28.12%	5.15%
BE17B2	533,462	479,697	53,765	71.91%	28.09%	5.18%
BE17B3	459,298	410,856	48,442	71.94%	28.06%	5.03%
BE17L1	444,914	405,784	39,130	72.19%	27.81%	5.32%
BE17L2	530,400	482,360	48,040	71.78%	28.22%	5.49%
BE17L3	458,580	416,890	41,690	72.03%	27.97%	5.09%
BE21B1	431,604	387,058	44,546	72.05%	27.95%	4.92%
BE21B2	468,195	420,675	47,520	71.82%	28.18%	4.96%
BE21B3	440,935	395,737	45,198	71.80%	28.20%	4.73%
BE21L1	437,821	400,429	37,392	72.14%	27.86%	5.25%
BE21L2	389,009	356,065	32,944	72.51%	27.49%	5.24%
BE21L3	347,547	315,990	31,557	72.68%	27.32%	5.79%
BE27B1	536,317	492,673	43,644	71.70%	28.30%	5.22%
BE27B2	634,028	574,484	59,544	71.12%	28.88%	4.74%
BE27B3	542,335	494,968	47,367	71.56%	28.44%	4.88%
BE27L1	406,931	374,739	32,192	72.26%	27.74%	4.27%
BE27L2	412,229	379,360	32,869	72.25%	27.75%	5.59%
BE27L3	446,540	410,485	36,055	71.98%	28.02%	5.50%
BE31B1	463,381	425,359	38,022	71.92%	28.08%	5.23%
BE31B2	475,408	426,377	49,031	71.77%	28.23%	5.28%
BE31B3	437,040	398,822	38,218	71.91%	28.09%	5.40%
BE31L1	293,728	264,407	29,321	72.66%	27.34%	5.07%
BE31L2	290,393	261,387	29,006	72.68%	27.32%	4.31%
BE31L3	356,589	317,875	38,714	72.17%	27.83%	5.23%
BE34B1	353,066	320,045	33,021	72.24%	27.76%	5.03%
BE34B2	66,777	60,706	6071	74.87%	25.13%	47.28%
BE34B3	466,221	416,752	49,469	71.79%	28.21%	4.87%
BE34L1	401,531	359,502	42,029	71.91%	28.09%	5.05%
BE34L2	347,704	312,392	35,312	72.09%	27.91%	5.00%
BE34L3	326,042	295,401	30,641	72.38%	27.62%	5.59%
BM6B1	76,822	69,735	7087	74.74%	25.26%	42.54%
BM6B2	76,472	70,083	6389	74.82%	25.18%	40.16%
BM6B3	114,505	104,733	9772	73.88%	26.12%	38.46%
BM6L1	23,381	20,572	2809	76.34%	23.66%	48.09%
BM6L2	66,117	60,659	5458	75.23%	24.77%	44.45%
BM6L3	85,270	77,902	7368	74.24%	25.76%	40.48%

SNP Number: Total numbers of SNPs; Genic SNP: Total numbers of SNPs in the genic region; Intergenic SNP: Total numbers of SNPs between genes; Transition: The percentage that the transition-type SNP accounts for all SNP locis; Transversion: The percentage that the transversion-type SNP loci accounts for all SNP sites; Heterozygosity: The percentage that the heterozygous SNPs account for all SNPs.

**Table 5:** Statistical results of differentially expressed genes.

DEGs	DEGnumber (newGene)	Up-Regulated (newGene)	Down-Regulated (newGene)
BE17B_vs_BE21B	410 (24)	218 (22)	192 (2)
BE17B_vs_BE27B	1958 (148)	1162 (138)	796 (10)
BE17B_vs_BE31B	1517 (108)	925 (101)	592 (7)
BE17B_vs_BE34B	1460 (79)	852 (73)	608 (6)
BE17B_vs_BM6B	5377 (339)	2580 (187)	2797 (152)
BE17L_vs_BE21L	655 (24)	371 (16)	284 (8)
BE17L_vs_BE27L	2866 (185)	1606 (148)	1260 (37)
BE17L_vs_BE31L	4413 (344)	2440 (295)	1973 (49)
BE17L_vs_BE34L	4326 (342)	2374 (299)	1952 (43)
BE17L_vs_BM6L	4560 (303)	2303 (168)	2257 (135)
BE17B_vs_BE17L	214 (13)	162 (6)	52 (7)
BE21B_vs_BE21L	1256 (194)	523 (20)	733 (174)
BE27B_vs_BE27L	195 (27)	51 (2)	144 (25)
BE31B_vs_BE31L	1226 (96)	606 (63)	620 (33)
BE34B_vs_BE34L	19 (3)	5 (1)	14 (2)
BM6B_vs_BM6L	104 (13)	58 (6)	46 (7)

**Table 6:** The most enriched GO terms.

DEGs	The Most Enriched GO Terms				
BE17B_vs_BE21B	regulation of calcium ion import	regulation of muscle filament sliding speed	regulation of euchromatin binding	dorsal root ganglion development	negative regulation of fibroblast growth factor receptor signaling pathway
BE17B_vs_BE27B	actin binding	motor activity	positive regulation of myoblast proliferation	cell division	positive regulation of cell proliferation
BE17B_vs_BE31B	striated muscle contraction	regulation of muscle filament sliding	skeletal muscle fiber development	muscle contraction	positive regulation of myoblast differentiation
BE17B_vs_BE34B	chordate embryonic development	regulation of cell cycle	positive regulation of fibroblast proliferation	muscle contraction	positive regulation of substrate-dependent cell migration
BE17B_vs_BM6B	translation	immune response	regulation of cell size	regulation of cell growth	regulation of G2/M transition mitotic cell cycle
BE17L_vs_BE21L	cell proliferation	muscle contraction	skeletal muscle fiber development	skeletal muscle tissus development	regulation of muscle filament sliding
BE17L_vs_BE27L	egulation of transcription involved in cell fate commitment	calcium-mediated signaling	glucose transport	signal transduction	Wnt signaling pathway, calcium modulating pathway
BE17L_vs_BE31L	cell maturation	embryonic limb morphogenesis	muscle contraction	chordate embryonic development	immune response
BE17L_vs_BE34L	embryonic hindlimb morphogenesis	positive regulation of protein process	regulation of actin cytoskeleton organization	positive regulation of cell proliferation	L-glutamate transmembrane transport
BE17L_vs_BM6L	Wnt receptor catabolic process	embryonic hindlimb morphogenesis	glucose transport	protein folding	immune response
BE17B_vs_BE17L	myoblast migration involved in skeletal muscle regeneration	positive regulation of glucocorticoid receptor signaling pathways	regulation of multicellular organism growth	cell adhesion	skeletal system development
BE21B_vs_BE21L	positive regulation of cellular process	metabolic process	fibroblast migration	RNA-dependent DNA biosynthetic process	positive regulation of cellular process
BE27B_vs_BE27L	positive regulation of MHC class I biosynthetic process	immune response	metabotic process	ubiquitin-dependent protein catabolic process	transmembrane transport
BE31B_vs_BE31L	regulation of cell shape	embryonic organ development	translation	muscle structure morphogenesis	DNA-dependent DNA replication
BE34B_vs_BE34L	skeletal muscle cell differentiation	skeletal muscle fiber adaptation	myotube differentiation involved in skeletal muscle regeneration	positive regulation of skeletal muscle tissue regeneration	protein phosphorylation
BM6B_vs_BM6L	muscle structure development	regulation of biological quality	myoblast fate commitment	embryonic skeletal joint morphogenesis	negative regulation of skeletal muscle tissue development

**Table 7:** Top 5 of KEGG enrichment.

DEGs	KEGG Enrichment				
BE17B_vs_BE21B	Focal adhesion	Regulation of actin cytoskeleton	MAPK signaling pathway	Wnt signaling pathway	ECM–receptor interaction
BE17B_vs_BE27B	Focal adhesion	Neuroactive ligand-receptor interaction	Purine metabolism	MAPK signaling pathway	Calcium signaling pathway
BE17B_vs_BE31B	Focal adhesion	MAPK signaling pathway	Purine metabolism	Cell cycle	Calcium signaling pathway
BE17B_vs_BE34B	Focal adhesion	MAPK signaling pathway	Neuroactive ligand–receptor interaction	Regulation of actin cytoskeleton	Endocytosis
BE17B_vs_BM6B	Leukocyte transendothelial migration	Thiamine metabolism	ErbB signaling pathway	Glucagon signaling pathway	RIG–I–like receptor signaling pathway
BE17L_vs_BE21L	Focal adhesion	Neuroactive ligand–receptor interaction	ECM–receptor interaction	MAPK signaling pathway	Regulation of actin cytoskeleton
BE17L_vs_BE27L	Neuroactive ligand–receptor interaction	Focal adhesion,	Calcium signaling pathway	MAPK signaling pathway	Oxidative phosphorylation
BE17L_vs_BE31L	Ribosome	Focal adhesion	Oxidative phosphorylation	MAPK signaling pathway	Regulation of actin cytoskeleton
BE17L_vs_BE34L	Focal adhesion	Neuroactive ligand–receptor interaction	Regulation of actin cytoskeleton	Oxidative phosphorylation	MAPK signaling pathway
BE17L_vs_BM6L	Neuroactive ligand–receptor interaction	MAPK signaling pathway	Oxidative phosphorylation	Focal adhesion	Calcium signaling pathway
BE17B_vs_BE17L	Focal adhesion	Neuroactive ligand–receptor interaction	ECM-receptor interaction	Cytokine–cytokine receptor interaction	Phagosome
BE21B_vs_BE21L	Focal adhesion	ECM–receptor interaction	Regulation of actin cytoskeleton	Phagosome	Neuroactive ligand–receptor interaction
BE27B_vs_BE27L	Glycerophospholipid metabolism	Glycerolipid metabolism	Tight junction	Cell adhesion molecules (CAMs)	Biosynthesis of amino acids
BE31B_vs_BE31L	Oxidative phosphorylation	Ribosome	Regulation of actin cytoskeleton	Calcium signaling pathway	MAPK signaling pathway
BE34B_vs_BE34L	Cell cycle	Endocytosis	MAPK signaling pathway	Cytokine–cytokine receptor interaction	Ubiquitin mediated proteolysis
BM6B_vs_BM6L	Adrenergic signaling in cardiomyocytes	Tight junction	Cardiac muscle contraction	MAPK signaling pathway	Apoptosis

## Data Availability

The datasets generated for this study can be found in the NCBI SRA (Submission: SUB8199414 and SUB8199659). Bioproject #PRJNA665025 and Biosamples #SAMN16238613-SAMN16238630, Bioproject #PRJNA665027 and Biosamples #SAMN16238633-SAMN16238650.

## Supplementary Materials

The following are available online at https://www.mdpi.com/2073-4425/11/10/1228/s1, Table S1 The Concentration and RIN value of sample RNA; Table S2 Statistics of sequence comparison between sample sequencing data and *Anas platyrhynchos* genome; Table S3 The five up- and down-regulated genes among the comparison samples; Table S4 Number of differentially expressed genes annotated; Figure S1 Volcano plot figure of DEGs in breast muscle; Figure S2 Volcano plot figure of DEGs in leg muscle; Figure S3 Volcano plot figure of DEGs in the comparison of breast and leg muscle; Figure S4 Cluster diagram of DEGs in breast muscle; Figure S5 Cluster diagram of DEGs in leg muscle; Figure S6 Cluster diagram of DEGs in the comparison of breast and leg muscle; Figure S7 COG classification results of DEGs in breast muscle; Figure S8 COG classification results of DEGs in leg muscle; Figure S9 COG classification results of DEGs in the comparison of breast and leg muscle; Figure S10 Signal pathway of focal adhesion; Figure S11 Signal pathway of MAPK; Figure S12 Signal pathway of regulation of the actin cytoskeleton.

## References

[1] Güller I., Russell A.P. (2010). MicroRNAs in skeletal muscle: Their role and regulation in development, disease and function. J. Physiol..

[2] Bi P., Ramirez-Martinez A., Li H., Cannavino J., McAnally J.R., Shelton J.M., Sánchez-Ortiz E., Bassel-Duby R., Olson E.N. (2017). Control of muscle formation by the fusogenic micropeptide myomixer. Science.

[3] Mitchell P.O., Mills S.T., Pavlath G.K. (2002). Calcineurin differentially regulates maintenance and growth of phenotypically distinct muscles. Am. J. Physiol. Physiol..

[4] Deries M., Thorsteinsdóttir S. (2016). Axial and limb muscle development: Dialogue with the neighbourhood. Cell. Mol. Life Sci..

[5] Scaal M., Marcelle C. (2018). Chick muscle development. Int. J. Dev. Biol..

[6] Buckingham M., Bajard L., Chang T., Daubas P., Hadchouel J., Meilhac S.M., Montarras D., Rocancourt D., Relaix F. (2003). The formation of skeletal muscle: From somite to limb. J. Anat..

[7] Guo B., Greenwood P.L., Cafe L.M., Zhou G., Zhang W., Dalrymple B.P. (2015). Transcriptome analysis of cattle muscle identifies potential markers for skeletal muscle growth rate and major cell types. BMC Genom..

[8] Hutton K.C., Vaughn M.A., Litta G., Turner B.J., Starkey J.D. (2014). Effect of vitamin D status improvement with 25-hydroxycholecalciferol on skeletal muscle growth characteristics and satellite cell activity in broiler chickens1,2. J. Anim. Sci..

[9] Costa V., Angelini C., De Feis I., Ciccodicola A. (2010). Uncovering the Complexity of Transcriptomes with RNA-Seq. J. Biomed. Biotechnol..

[10] Waern K., Nagalakshmi U., Snyder M. (2011). RNA sequencing. Methods Mol. Biol..

[11] Trapnell C., Roberts A., Goff L., Pertea G., Kim D., Kelley D.R., Pimentel H., Salzberg S.L., Rinn J.L., Pachter L. (2012). Differential gene and transcript expression analysis of RNA-seq experiments with TopHat and Cufflinks. Nat. Protoc..

[12] Kumar D., Bansal G., Narang A., Basak T., Abbas T., Dash D. (2016). Integrating transcriptome and proteome profiling: Strategies and applications. Proteomics.

[13] Xue Q., Zhang G., Li T., Ling J., Zhang X., Wang J. (2017). Transcriptomic profile of leg muscle during early growth in chicken. PLoS ONE.

[14] Zhang Z., Du H., Yang C., Li Q., Qiu M., Song X., Yu C., Jiang X., Liu L., Hu C. (2019). Comparative transcriptome analysis reveals regulators mediating breast muscle growth and development in three chicken breeds. Anim. Biotechnol..

[15] Xu T.-S., Gu L.-H., Huang W., Xia W.-L., Zhang Y.-S., Zhang Y.-G., Rong G., Schachtschneider K.M., Hou S.-S. (2017). Gene expression profiling in Pekin duck embryonic breast muscle. PLoS ONE.

[16] Zhu C., Song W., Tao Z., Liu H., Xu W., Zhang S., Li H. (2017). Deep RNA sequencing of pectoralis muscle transcriptomes during late-term embryonic to neonatal development in indigenous Chinese duck breeds. PLoS ONE.

[17] Liu H.X., Hu Y., Ji G.G., Li H.F. (2014). Rapid-Sexing Poultries via a New Pair of Universal Primers. J. Agr. Biotechnol..

[18] Mutz K.-O., Heilkenbrinker A., Lönne M., Walter J.-G., Stahl F. (2013). Transcriptome analysis using next-generation sequencing. Curr. Opin. Biotechnol..

[19] Adiconis X., Borges-Rivera D., Satija R., DeLuca D.S., Busby M.A., Berlin A.M., Sivachenko A., Thompson D.A., Wysoker A., Fennell T. (2013). Comparative analysis of RNA sequencing methods for degraded or low-input samples. Nat. Methods.

[20] Bihan-Duval E.L., Millet N., Remignon H. (1999). Broiler meat quality: Effect of selection for increased carcass quality and estimates of genetic parameters. Poult. Sci..

[21] Sachidanandam R., Weissman D., Schmidt S.C., Kakol J.M., Stein L.D., Marth G., Sherry S., Mullikin J.C., Mortimore B.J., Willey D.L. (2001). A map of human genome sequence variation containing 1.42 million single nucleotide polymorphisms. Nature.

[22] Huang Y., Zou Y., He H., Dang Y.-L., Qi X.-S., Chen H., Lin Q., Zheng L., Zhang Z.-J., Lei C. (2017). Effects of genetic variants of the bovine WNT8A gene on nine important growth traits in beef cattle. J. Genet..

[23] Wu S., Wang Y., Ning Y., Guo H., Xiaoyu W., Zhang L., Khan R., Cheng G., Wang H., Zan L.S. (2018). Genetic Variants in STAT3 Promoter Regions and Their Application in Molecular Breeding for Body Size Traits in Qinchuan Cattle. Int. J. Mol. Sci..

[24] Sun Z., Bhagwate A., Prodduturi N., Yang P., Kocher J.-P.A. (2017). Indel detection from RNA-seq data: Tool evaluation and strategies for accurate detection of actionable mutations. Brief. Bioinform..

[25] Yang R., Van Etten J.L., Dehm S.M. (2018). Indel detection from DNA and RNA sequencing data with transIndel. BMC Genom..

[26] Chen L., Tovar-Corona J.M., Urrutia A.O. (2012). Alternative Splicing: A Potential Source of Functional Innovation in the Eukaryotic Genome. Int. J. Evol. Biol..

[27] Kornblihtt A.R., Schor I.E., Alló M., Dujardin G., Petrillo E., Muñoz M.J. (2013). Alternative splicing: A pivotal step between eukaryotic transcription and translation. Nat. Rev. Mol. Cell Biol..

[28] Traunmuller L., Gomez A.M., Nguyen T.M., Scheifele P. (2016). Control of neuronal synapse specifcation by a highly dedicated alternative splicing program. Science.

[29] Schwerk C., Schulze-Osthoff K. (2005). Regulation of Apoptosis by Alternative Pre-mRNA Splicing. Mol. Cell.

[30] Thorsteinsdóttir S., Roelen B.A.J., Goumans M.-J., Oostwaard D.W.-V., Gaspar A.C., Mummery C. (1999). Expression of the α6A integrin splice variant in developing mouse embryonic stem cell aggregates and correlation with cardiac muscle differentiation. Differentiation.

[31] Schmucker D., Clemens J.C., Shu H., Worby C., Xiao J., Muda M., Dixon J.E., Zipursky S. (2000). Drosophila Dscam Is an Axon Guidance Receptor Exhibiting Extraordinary Molecular Diversity. Cell.

[32] Gueroussov S., Gonatopoulos-Pournatzis T., Irimia M., Raj B., Lin Z.-Y., Gingras A., Blencowe B.J. (2015). An alternative splicing event amplifies evolutionary differences between vertebrates. Science.

[33] Yin Z., Zhang F., Smith J., Kuo R., Hou Z.-C. (2019). Full-length transcriptome sequencing from multiple tissues of duck, Anas platyrhynchos. Sci. Data.

[34] Park M.Y., Jang H.D., Lee S.Y., Lee K.J., Kim E. (2004). Fas-associated factor-1 inhibits nuclear factor-kappaB (NF-kappaB) activity by interfering with nuclear translocation of the RelA (p65) subunit of NF-kappaB. J. Biol. Chem..

[35] Park M.Y., Moon J.H., Lee K.S., Choi H.I., Chung J., Hong H.J., Kim E. (2007). FAF1 suppresses IkappaB kinase (IKK) activation by disrupting the IKK complex assembly. J. Biol. Chem..

[36] Menges C.W., Altomare D.A., Testa J.R. (2009). FAS-associated factor 1 (FAF1): Diverse functions and implications for oncogenesis. Cell Cycle.

[37] Song E.J., Yim S.-H., Kim E., Kim N.-S., Lee K.J. (2005). Human Fas-Associated Factor 1, Interacting with Ubiquitinated Proteins and Valosin-Containing Protein, Is Involved in the Ubiquitin-Proteasome Pathway. Mol. Cell. Biol..

[38] Jang M.-S., Sul J.-W., Choi B.-J., Lee S.-J., Suh J.-H., Kim N.-S., Kim W.H., Lim D.-S., Lee C.-W., Kim E.-H. (2008). Negative Feedback Regulation of Aurora-A via Phosphorylation of Fas-associated Factor-1. J. Biol. Chem..

[39] Zhang L., Zhou F., Van Laar T., Zhang J., van Dam H., Dijke P.T. (2011). Fas-associated factor 1 antagonizes Wnt signaling by promoting β-catenin degradation. Mol. Biol. Cell.

[40] Steinhart Z., Angers S. (2018). Wnt signaling in development and tissue homeostasis. Development.

[41] Rudnicki M.A., Williams B.O. (2015). Wnt signaling in bone and muscle. Bone.

[42] Adham I.M., Khulan J., Held T., Schmidt B., Meyer B.I., Meinhardt A., Engel W. (2008). Fas-associated factor (FAF1) is required for the early cleavage-stages of mouse embryo. Mol. Hum. Reprod..

[43] Ryu S.-W., Chae S.-K., Lee K.J., Kim E. (1999). Identification and Characterization of Human Fas Associated Factor 1, hFAF1. Biochem. Biophys. Res. Commun..

[44] Fröhlich T., Risau W., Flamme I. (1998). Characterization of novel nuclear targeting and apoptosis-inducing domains in FAS associated factor 1. J. Cell Sci..

[45] Siderovski D.P., Willard F.S. (2005). The GAPs, GEFs, and GDIs of heterotrimeric G-protein alpha subunits. Int. J. Biol. Sci..

[46] Berman D.M., Kozasa T., Gilman A.G. (1996). The GTPase-activating Protein RGS4 Stabilizes the Transition State for Nucleotide Hydrolysis. J. Biol. Chem..

[47] Sjögren B., Blazer L.L., Neubig R.R. (2010). Regulators of G Protein Signaling Proteins as Targets for Drug Discovery. Prog. Mol. Biol. Transl. Sci..

[48] Benians A., Nobles M., Hosny S., Tinker A. (2005). Regulators of G-protein Signaling Form a Quaternary Complex with the Agonist, Receptor, and G-protein. J. Biol. Chem..

[49] Bansal G., Druey K.M., Xie Z. (2007). R4 RGS proteins: Regulation of G-protein signaling and beyond. Pharmacol. Ther..

[50] De Vries L. (1999). RGS proteins: More than just GAPs for heterotrimeric G proteins. Trends Cell Biol..

[51] Yu Y., Yoon S.-O., Poulogiannis G., Yang Q., Ma X.M., Villén J., Kubica N., Hoffman G.R., Cantley L.C., Gygi S.P. (2011). Phosphoproteomic Analysis Identifies Grb10 as an mTORC1 Substrate That Negatively Regulates Insulin Signaling. Science.

[52] Liu B., Liu F. (2014). Feedback regulation of mTORC1 by Grb10 in metabolism and beyond. Cell Cycle.

[53] Smith F.M., Holt L.J., Garfield A.S., Charalambous M., Koumanov F., Perry M., Bazzani R., Sheardown S.A., Hegarty B.D., Lyons R.J. (2007). Mice with a Disruption of the Imprinted Grb10 Gene Exhibit Altered Body Composition, Glucose Homeostasis, and Insulin Signaling during Postnatal Life. Mol. Cell. Biol..

[54] Deng Y., Zhang M., Riedel H. (2008). Mitogenic roles of Gab1 and Grb10 as direct cellular partners in the regulation of MAP kinase signaling. J. Cell. Biochem..

[55] Holt L.J., Siddle K. (2005). Grb10 and Grb14: Enigmatic regulators of insulin action–and more?. Biochem. J..

[56] Desbuquois B., Carré N., Burnol A.F. (2013). Regulation of insulin and type 1 insulin-like growth factor signaling and action by the Grb10/14 and SH2B1/B2 adaptor proteins. FEBS J..

[57] Jahn T., Seipel P., Urschel S., Peschel C., Duyster J. (2002). Role for the Adaptor Protein Grb10 in the Activation of Akt. Mol. Cell. Biol..

[58] Langlais P., Dong L.Q., Ramos F.J., Hu D., Li Y., Quon M.J., Liu F. (2004). Negative Regulation of Insulin-Stimulated Mitogen-Activated Protein Kinase Signaling By Grb10. Mol. Endocrinol..

[59] Monami G., Emiliozzi V., Morrione A. (2008). Grb10/Nedd4-mediated multiubiquitination of the insulin-like growth factor receptor regulates receptor internalization. J. Cell Physiol..

[60] Wang L., Balas B., Christ-Roberts C.Y., Kim R.Y., Ramos F.J., Kikani C.K., Li C., Deng C., Reyna S., Musi N. (2007). Peripheral Disruption of the Grb10 Gene Enhances Insulin Signaling and Sensitivity In Vivo. Mol. Cell. Biol..

[61] Verbrugge S.A.J., Schönfelder M., Becker L., Nezhad F.Y., De Angelis M.H., Wackerhage H. (2018). Genes Whose Gain or Loss-Of-Function Increases Skeletal Muscle Mass in Mice: A Systematic Literature Review. Front. Physiol..

[62] Du S.J., Tan X., Zhang J. (2014). SMYD Proteins: Key Regulators in Skeletal and Cardiac Muscle Development and Function. Anat. Rec. Adv. Integr. Anat. Evol. Biol..

[63] Kristin L., Mark B. (2011). SET/MYND Lysine Methyltransferases Regulate Gene Transcription and Protein Activity. Genes.

[64] Fujii T., Tsunesumi S.I., Yamaguchi K., Watanabe S., Furukawa Y. (2011). Smyd3 Is Required for the Development of Cardiac and Skeletal Muscle in Zebrafish. PLoS ONE.

[65] Tracy C.M., Warren J.S., Szulik M., Wang L., Garcia J., Makaju A., Russell K., Miller M., Franklin S. (2018). The Smyd family of methyltransferases: Role in cardiac and skeletal muscle physiology and pathology. Curr. Opin. Physiol..

[66] Proserpio V., Fittipaldi R., Ryall J.G., Sartorelli V., Caretti G. (2013). The methyltransferase SMYD3 mediates the recruitment of transcriptional cofactors at the myostatin and c-Met genes and regulates skeletal muscle atrophy. Genes Dev..

[67] Palstra A.P., Tudorache C., Rovira M., Brittijn S.A., Burgerhout E., Thillart G.E.E.J.M.V.D., Spaink H.P., Planas J.V. (2010). Establishing Zebrafish as a Novel Exercise Model: Swimming Economy, Swimming-Enhanced Growth and Muscle Growth Marker Gene Expression. PLoS ONE.

[68] Zhang L., Zhou Y., Wu W., Hou L., Chen H., Zuo B., Xiong Y., Yang J. (2017). Skeletal Muscle-Specific Overexpression of PGC-1α Induces Fiber-Type Conversion through Enhanced Mitochondrial Respiration and Fatty Acid Oxidation in Mice and Pigs. Int. J. Biol. Sci..

[69] Barton P.J.R., Townsend P.J., Brand N.J., Yacoub M.H. (1997). Localization of the fast skeletal muscle troponin I gene (TNNI2) to 11p15.5: Genes for troponin I and T are organized in pairs. Ann. Hum. Genet..

[70] Sheng J.-J., Jin J.-P. (2016). TNNI1, TNNI2 and TNNI3: Evolution, regulation, and protein structure–function relationships. Gene.

[71] Zhu X., Wang F., Zhao Y., Yang P., Chen J., Sun H., Liu L., Li W., Pan L., Guo Y. (2014). A Gain-of-Function Mutation in Tnni2 Impeded Bone Development through Increasing Hif3a Expression in DA2B Mice. PLoS Genet..

[72] Farah C.S., Miyamoto C., Ramos C.H., Da Silva A.C., Quaggio R.B., Fujimori K., Smillie L.B., Reinach F.C. (1994). Structural and regulatory functions of the NH2- and COOH-terminal regions of skeletal muscle troponin I. J. Biol. Chem..

[73] Yoshimoto Y., Ikemoto-Uezumi M., Hitachi K., Fukada S.-I., Uezumi A. (2020). Methods for Accurate Assessment of Myofiber Maturity During Skeletal Muscle Regeneration. Front. Cell Dev. Biol..

[74] Vigoreaux J.O. (2001). Genetics of theDrosophila flight muscle myofibril: A window into the biology of complex systems. BioEssays.

[75] Zhu L., Lyons G.E., Juhasz O., Joya J.E., Hardeman E.C., Wade R. (1995). Developmental Regulation of Troponin I Isoform Genes in Striated Muscles of Transgenic Mice. Dev. Biol..

[76] Duperret E.K., Ridky T.W. (2013). Focal adhesion complex proteins in epidermis and squamous cell carcinoma. Cell Cycle.

[77] Graham Z.A., Gallagher P.M., Cardozo C.P. (2015). Focal adhesion kinase and its role in skeletal muscle. J. Muscle Res. Cell Motil..

[78] Wagner E.F., Nebreda Á.R. (2009). Signal integration by JNK and p38 MAPK pathways in cancer development. Nat. Rev. Cancer.

[79] Ito N., Ruegg U.T., Takeda S. (2018). ATP-Induced Increase in Intracellular Calcium Levels and Subsequent Activation of mTOR as Regulators of Skeletal Muscle Hypertrophy. Int. J. Mol. Sci..

[80] Desmarais V., Ghosh M., Eddy R., Condeelis J.S. (2004). Cofilin takes the lead. J. Cell Sci..

[81] Le Clainche C., Carlier M.-F. (2008). Regulation of Actin Assembly Associated With Protrusion and Adhesion in Cell Migration. Physiol. Rev..

[82] Tang D.D., Gerlach B.D. (2017). The roles and regulation of the actin cytoskeleton, intermediate filaments and microtubules in smooth muscle cell migration. Respir. Res..

