# Supplementary material for: Comparative Transcriptome Profiling of Skeletal Muscle from Black Muscovy Duck at Different Growth Stages Using RNA-seq
Source: Genes (Basel). 2020 Oct 20;11(10):1228. doi: 10.3390/genes11101228 (PMC7590229; doi:10.3390/genes11101228)
Supplement: Supplementary file 1 [file genes-11-01228-s001.zip › Supplementary Files/Table S1.docx]

**Table S1 The Concentration and RIN value of sample RNA**

| Chest Muscle | Concentration (ng/μL) | RIN value | Leg Muscle | Concentration (ng/μL) | RIN value |
| --- | --- | --- | --- | --- | --- |
| BE17B1 | 243.9 | 8.2 | BE17L1 | 365.8 | 8.5 |
| BE17B2 | 371.7 | 9.9 | BE17L2 | 848.4 | 9.0 |
| BE17B3 | 256.6 | 7.9 | BE17L3 | 1960.7 | 8.9 |
| BE21B1 | 778.2 | 9.3 | BE21L1 | 782.2 | 9.0 |
| BE21B2 | 688.1 | 8.8 | BE21L2 | 953.3 | 8.9 |
| BE21B3 | 732.7 | 9.2 | BE21L3 | 1370.7 | 8.8 |
| BE27B1 | 384.0 | 8.5 | BE27L1 | 247.6 | 7.8 |
| BE27B2 | 2841.6 | 8.1 | BE27L2 | 469.2 | 8.4 |
| BE27B3 | 125.2 | 9.5 | BE27L3 | 340.9 | 8.3 |
| BE31B1 | 520.9 | 8.1 | BE31L1 | 968.3 | 7.8 |
| BE31B2 | 417.2 | 7.7 | BE31L2 | 889.4 | 7.9 |
| BE31B3 | 1010.5 | 8.2 | BE31L3 | 872.2 | 8.0 |
| BE34B1 | 685.2 | 7.7 | BE34L1 | 492.1 | 7.8 |
| BE34B2 | 659.2 | 8.4 | BE34L2 | 673.6 | 7.9 |
| BE34B3 | 550.4 | 8.2 | BE34L3 | 441.4 | 8.0 |
| BM6B1 | 355.8 | 8.5 | BM6L1 | 539.5 | 8.6 |
| BM6B2 | 297.1 | 8.7 | BM6L2 | 380.6 | 8.6 |
| BM6B3 | 240.9 | 8.5 | BM6L3 | 330.2 | 8.6 |
