# Supplementary material for: Comparative Transcriptome Profiling of Skeletal Muscle from Black Muscovy Duck at Different Growth Stages Using RNA-seq
Source: Genes (Basel). 2020 Oct 20;11(10):1228. doi: 10.3390/genes11101228 (PMC7590229; doi:10.3390/genes11101228)
Supplement: Supplementary file 1 [file genes-11-01228-s001.zip › Supplementary Files/Table S2.docx]

**Table S2 Statistics of sequence comparison between sample sequencing data and reference genome.**

| Samples | Total Reads | Mapped Reads | Uniq Mapped Reads | Multiple Map Reads | Reads Map to '＋' | Reads Map to '－' |
| --- | --- | --- | --- | --- | --- | --- |
| BE17B1 | 53,528,944 | 33,616,239 (62.80%) | 31,933,968 (59.66%) | 1,682,271 (3.14%) | 16,695,292 (31.19%) | 16,819,748 (31.42%) |
| BE17B2 | 64,049,872 | 40,303,805 (62.93%) | 38,405,875 (59.96%) | 1,897,930 (2.96%) | 20,010,754 (31.24%) | 20,163,529 (31.48%) |
| BE17B3 | 47,188,640 | 34,394,591 (72.89%) | 30,608,915 (64.87%) | 3,785,676 (8.02%) | 16,344,899 (34.64%) | 16,980,416 (35.98%) |
| BE17L1 | 53,101,146 | 34,264,357 (64.53%) | 32,617,322 (61.42%) | 1,647,035 (3.10%) | 17,027,927 (32.07%) | 17,130,323 (32.26%) |
| BE17L2 | 66,211,580 | 41,885,913 (63.26%) | 39,902,374 (60.26%) | 1,983,539 (3.00%) | 20,801,712 (31.42%) | 20,972,513 (31.67%) |
| BE17L3 | 44,467,622 | 27,534,027 (61.92%) | 26,236,604 (59.00%) | 1,297,423 (2.92%) | 13,666,579 (30.73%) | 13,775,339 (30.98%) |
| BE21B1 | 42,713,632 | 31,327,289 (73.34%) | 28,069,069 (65.71%) | 3,258,220 (7.63%) | 14,972,432 (35.05%) | 15,440,811 (36.15%) |
| BE21B2 | 46,046,128 | 34,092,922 (74.04%) | 30,829,307 (66.95%) | 3,263,615 (7.09%) | 16,336,231 (35.48%) | 16,828,925 (36.55%) |
| BE21B3 | 44,464,314 | 31,764,342 (71.44%) | 28,638,328 (64.41%) | 3,126,014 (7.03%) | 15,230,090 (34.25%) | 15,642,976 (35.18%) |
| BE21L1 | 54,467,886 | 35,117,080 (64.47%) | 33,258,051 (61.06%) | 1,859,029 (3.41%) | 17,454,836 (32.05%) | 17,559,919 (32.24%) |
| BE21L2 | 47,642,322 | 30,618,314 (64.27%) | 29,033,900 (60.94%) | 1,584,414 (3.33%) | 15,211,712 (31.93%) | 15,314,786 (32.15%) |
| BE21L3 | 50,504,848 | 30,699,205 (60.78%) | 28,977,233 (57.38%) | 1,721,972 (3.41%) | 15,227,694 (30.15%) | 15,376,609 (30.45%) |
| BE27B1 | 52,010,038 | 31,543,727 (60.65%) | 29,755,236 (57.21%) | 1,788,491 (3.44%) | 15,682,052 (30.15%) | 15,788,606 (30.36%) |
| BE27B2 | 53,805,826 | 40,123,664 (74.57%) | 35,410,565 (65.81%) | 4,713,099 (8.76%) | 19,117,679 (35.53%) | 19,792,419 (36.78%) |
| BE27B3 | 48,452,782 | 36,053,248 (74.41%) | 31,577,871 (65.17%) | 4,475,377 (9.24%) | 17,194,827 (35.49%) | 17,787,604 (36.71%) |
| BE27L1 | 47,269,414 | 28,867,801 (61.07%) | 26,971,272 (57.06%) | 1,896,529 (4.01%) | 14,342,178 (30.34%) | 14,470,904 (30.61%) |
| BE27L2 | 51,122,998 | 32,021,848 (62.64%) | 30,026,632 (58.73%) | 1,995,216 (3.90%) | 15,915,337 (31.13%) | 16,042,516 (31.38%) |
| BE27L3 | 58,683,002 | 35,326,029 (60.20%) | 33,038,735 (56.30%) | 2,287,294 (3.90%) | 17,531,225 (29.87%) | 17,731,349 (30.22%) |
| BE31B1 | 43,416,768 | 26,201,608 (60.35%) | 24,834,644 (57.20%) | 1,366,964 (3.15%) | 13,011,232 (29.97%) | 13,125,531 (30.23%) |
| BE31B2 | 42,090,182 | 29,394,974 (69.84%) | 25,586,435 (60.79%) | 3,808,539 (9.05%) | 13,735,393 (32.63%) | 14,408,459 (34.23%) |
| BE31B3 | 47,332,274 | 29,174,700 (61.64%) | 27,428,169 (57.95%) | 1,746,531 (3.69%) | 14,484,469 (30.60%) | 14,611,374 (30.87%) |
| BE31L1 | 43,547,570 | 30,007,810 (68.91%) | 22,902,847 (52.59%) | 7,104,963 (16.32%) | 12,767,274 (29.32%) | 14,244,399 (32.71%) |
| BE31L2 | 39,401,532 | 27,078,362 (68.72%) | 21,975,685 (55.77%) | 5,102,677 (12.95%) | 12,042,060 (30.56%) | 13,086,584 (33.21%) |
| BE31L3 | 50,134,796 | 35,578,192 (70.97%) | 29,525,496 (58.89%) | 6,052,696 (12.07%) | 16,123,246 (32.16%) | 17,296,350 (34.50%) |
| BE34B1 | 39,941,138 | 28,658,110 (71.75%) | 23,030,485 (57.66%) | 5,627,625 (14.09%) | 12,762,734 (31.95%) | 13,875,420 (34.74%) |
| BE34B2 | 43,282,988 | 35,507,459 (82.04%) | 30,289,226 (69.98%) | 5,218,233 (12.06%) | 13,482,011 (31.15%) | 16,380,642 (37.85%) |
| BE34B3 | 48,495,186 | 34,138,137 (70.39%) | 29,745,432 (61.34%) | 4,392,705 (9.06%) | 15,976,635 (32.94%) | 16,717,189 (34.47%) |
| BE34L1 | 39,650,692 | 27,868,329 (70.28%) | 24,318,991 (61.33%) | 3,549,338 (8.95%) | 13,024,948 (32.85%) | 13,653,806 (34.44%) |
| BE34L2 | 41,245,142 | 27,565,935 (66.83%) | 23,667,106 (57.38%) | 3,898,829 (9.45%) | 12,795,294 (31.02%) | 13,419,955 (32.54%) |
| BE34L3 | 43,008,582 | 29,535,007 (68.67%) | 22,428,157 (52.15%) | 7,106,850 (16.52%) | 12,581,022 (29.25%) | 14,170,686 (32.95%) |
| BM6B1 | 55,777,854 | 45,396,620 (81.39%) | 34,392,152 (61.66%) | 11,004,468 (19.73%) | 13,918,490 (24.95%) | 19,781,791 (35.47%) |
| BM6B2 | 52,631,408 | 43,974,737 (83.55%) | 34,796,699 (66.11%) | 9,178,038 (17.44%) | 13,354,243 (25.37%) | 19,062,298 (36.22%) |
| BM6B3 | 64,283,852 | 51,021,073 (79.37%) | 37,246,526 (57.94%) | 13,774,547 (21.43%) | 14,805,988 (23.03%) | 21,955,456 (34.15%) |
| BM6L1 | 45,135,658 | 30,264,877 (67.05%) | 23,077,005 (51.13%) | 7,187,872 (15.93%) | 9,449,302 (20.94%) | 13,271,831 (29.40%) |
| BM6L2 | 50,661,720 | 40,201,634 (79.35%) | 28,272,203 (55.81%) | 11,929,431 (23.55%) | 9,228,720 (18.22%) | 16,451,610 (32.47%) |
| BM6L3 | 52,541,572 | 43,490,607 (82.77%) | 33,330,555 (63.44%) | 10,160,052 (19.34%) | 12,111,818 (23.05%) | 18,468,244 (35.15%) |

Note: Uniq Mapped Reads: number of Reads aligned to only one position in the reference genome and the percentage of Clean Reads; Multiple Map Reads: number of Reads aligned to two or more locations in the reference genome and the percentage of Clean Reads; Reads Map to '＋': number of Reads aligned to the positive strand in the reference genome and the percentage of Clean Reads; Reads Map to '－': number of reads aligned to the negative strand in the reference genome and the percentage of Clean Reads.
