# Supplementary material for: Comparative Transcriptome Profiling of Skeletal Muscle from Black Muscovy Duck at Different Growth Stages Using RNA-seq
Source: Genes (Basel). 2020 Oct 20;11(10):1228. doi: 10.3390/genes11101228 (PMC7590229; doi:10.3390/genes11101228)
Supplement: Supplementary file 1 [file genes-11-01228-s001.zip › Supplementary Files/Table S3.docx]

**Table S3 The five up- and down-regulated genes among the comparison samples**

| DEGs | up-regulated | down-regulated |
| --- | --- | --- |
| BE17B_vs_BE21B | *SOX*6, *LGALS*8, *RPS27A*, *ADAM*23, *RNF*122 | *TP*73, *IL4R*, *USP*49, *LOC113844753*, *CPA*6 |
| BE17B_vs_BE27B | *RILPL*1, *PURG*, *FKBP*3, *SLC4A*4, *ABCA*2 | *GAP*43, *STK32B*, *PENK*, *GDPD*4, *MLNR* |
| BE17B_vs_BE31B | *RPS6KA*3, *LOC101800677*, *ZNF*703, *MIPOL*1, *LAMA*4 | *NDUFS*7*, PENK, LOC113845763, STK32B*, *LOC101798073* |
| BE17B_vs_BE34B | *MRPS*2*, GNB*5*, GLRX*3*, RMND*1*, FNIP*1 | *GAP*43*, LOC101802910, PENK, STK32B, TP*73 |
| BE17B_vs_BM6B | *PDETA, PHF*3*, MDH*1*, SLIRP, R3HDM*1 | *LOC101803605, STRA*6*, TPBG, CRABP*1*, MYL*4 |
| BE17L_vs_BE21L | *VEGFA, SH3TC*1*, SOX*17*, FKBP*3*, AGL* | *MATN*1*, COL9A*1*, PIGW, LOC113840466, COL9A*3 |
| BE17L_vs_BE27L | *ASRGL*1*, MSRA, SLC9A3R*2*, ATP5MC*3*, FBXL*4 | *TTC30B, MATN*1*, LHX*9*, TBX*5*, EPYC* |
| BE17L_vs_BE31L | *NPTX*2*, LOC113839804,* newGene_5042*, CUTC, ADGRG*1 | *TPBG, LOC113840809, MTF*1*, MAML*1*, CPLX1* |
| BE17L_vs_BE34L | *SUGP*1*, TIAL*1*, LOC101792248, RDM*1*, PDXK* | *TPBG, MTF*1*, SETD*2*, MAML*1*, RSPO*2 |
| BE17L_vs_BM6L | *TMEM*189*, AZI*2*, COPS*2*, NMD*3*, GLYCTK* | *CRABP*1*, FBN*3*, ZPLD*1*, TPBG, LHX*9 |
| BE17B_vs_BE17L | *TENM*3*, CCDC*80*, POU2AF*1*, CSGALNACT*1*, GDF*7 | *DIO*3*, RBM*20*, CNNM*1*, HPSE*2*, UBE2C* |
| BE21B_vs_BE21L | *LOC101795565, KCNAB*1*, TRAM*1*, HAS*2*, SLCO3A*1 | newGene_33850*,* newGene_31533*,* newGene_31534*,* newGene_28667*, JHY* |
| BE27B_vs_BE27L | *IARS, PIGY, ABI3BP, LDB*3*, ICA*1 | *TTC30B, LOC113843568,* newGene_2437*,* newGene_9000*, PTRH*2 |
| BE31B_vs_BE31L | *NOP*56*, PIN*4*, LOC101795065, ABHD*11*, KLHDC*4 | *SLC8A*3*, TMEM*222*, LOC113840809, DIO*3*, NEURL1B* |
| BE34B_vs_BE34L | *FNBP*4*, IL17RD, FAM53B,* newGene_24580*, LRIT*2 | *CIQTNF*5*,* newGene_16088*,* newGene_19459*, LOC113840453, C20H19orf*24 |
| BM6B_vs_BM6L | *PRSS*23*, RGS*2*, SERINC*2*, MFAP3L, VSNL*1 | newGene_20619, *LOC113843303*, *FRMD*5, *LOC101795831*, newGene_36986 |
