# Supplementary material for: Comparative Transcriptome Profiling of Skeletal Muscle from Black Muscovy Duck at Different Growth Stages Using RNA-seq
Source: Genes (Basel). 2020 Oct 20;11(10):1228. doi: 10.3390/genes11101228 (PMC7590229; doi:10.3390/genes11101228)
Supplement: Supplementary file 1 [file genes-11-01228-s001.zip › Supplementary Files/Table S4.docx]

**Table S4 Number of differentially expressed genes annotated**

| DEGs | Total | COG | GO | KEGG | KOG | NR | Pfam | Swiss-Prot | eggNOG |
| --- | --- | --- | --- | --- | --- | --- | --- | --- | --- |
| BE17B_vs_BE21B | 397 | 120 | 319 | 261 | 293 | 395 | 365 | 301 | 389 |
| BE17B_vs_BE27B | 1890 | 630 | 1517 | 1282 | 1380 | 1880 | 1741 | 1438 | 1830 |
| BE17B_vs_BE31B | 1464 | 501 | 1169 | 986 | 1057 | 1456 | 1350 | 1091 | 1419 |
| BE17B_vs_BE34B | 1432 | 481 | 1156 | 971 | 1032 | 1426 | 1322 | 1065 | 1394 |
| BE17B_vs_BM6B | 5253 | 1775 | 4191 | 3542 | 3788 | 5231 | 4816 | 3788 | 5118 |
| BE17L_vs_BE21L | 641 | 205 | 511 | 422 | 444 | 638 | 603 | 505 | 626 |
| BE17L_vs_BE27L | 2771 | 939 | 2227 | 1886 | 1969 | 2759 | 2570 | 2069 | 2696 |
| BE17L_vs_BE31L | 4272 | 1459 | 3418 | 2887 | 3086 | 4251 | 3866 | 2999 | 4149 |
| BE17L_vs_BE34L | 4173 | 1385 | 3350 | 2821 | 3011 | 4154 | 3773 | 2987 | 4049 |
| BE17L_vs_BM6L | 4427 | 1491 | 3565 | 2974 | 3170 | 4407 | 4071 | 3212 | 4301 |
| BE17B_vs_BE17L | 207 | 56 | 169 | 133 | 148 | 207 | 199 | 168 | 203 |
| BE21B_vs_BE21L | 1175 | 348 | 893 | 748 | 796 | 1172 | 1008 | 803 | 1115 |
| BE27B_vs_BE27L | 182 | 49 | 140 | 111 | 115 | 182 | 157 | 125 | 171 |
| BE31B_vs_BE31L | 1184 | 397 | 956 | 806 | 856 | 1181 | 1078 | 815 | 1147 |
| BE34B_vs_BE34L | 17 | 3 | 14 | 9 | 10 | 17 | 14 | 12 | 15 |
| BM6B_vs_BM6L | 96 | 30 | 74 | 66 | 76 | 96 | 86 | 72 | 92 |
