# Supplementary figures and images for: Comparative Transcriptome Profiling of Skeletal Muscle from Black Muscovy Duck at Different Growth Stages Using RNA-seq
Source: Genes (Basel). 2020 Oct 20;11(10):1228. doi: 10.3390/genes11101228 (PMC7590229; doi:10.3390/genes11101228)

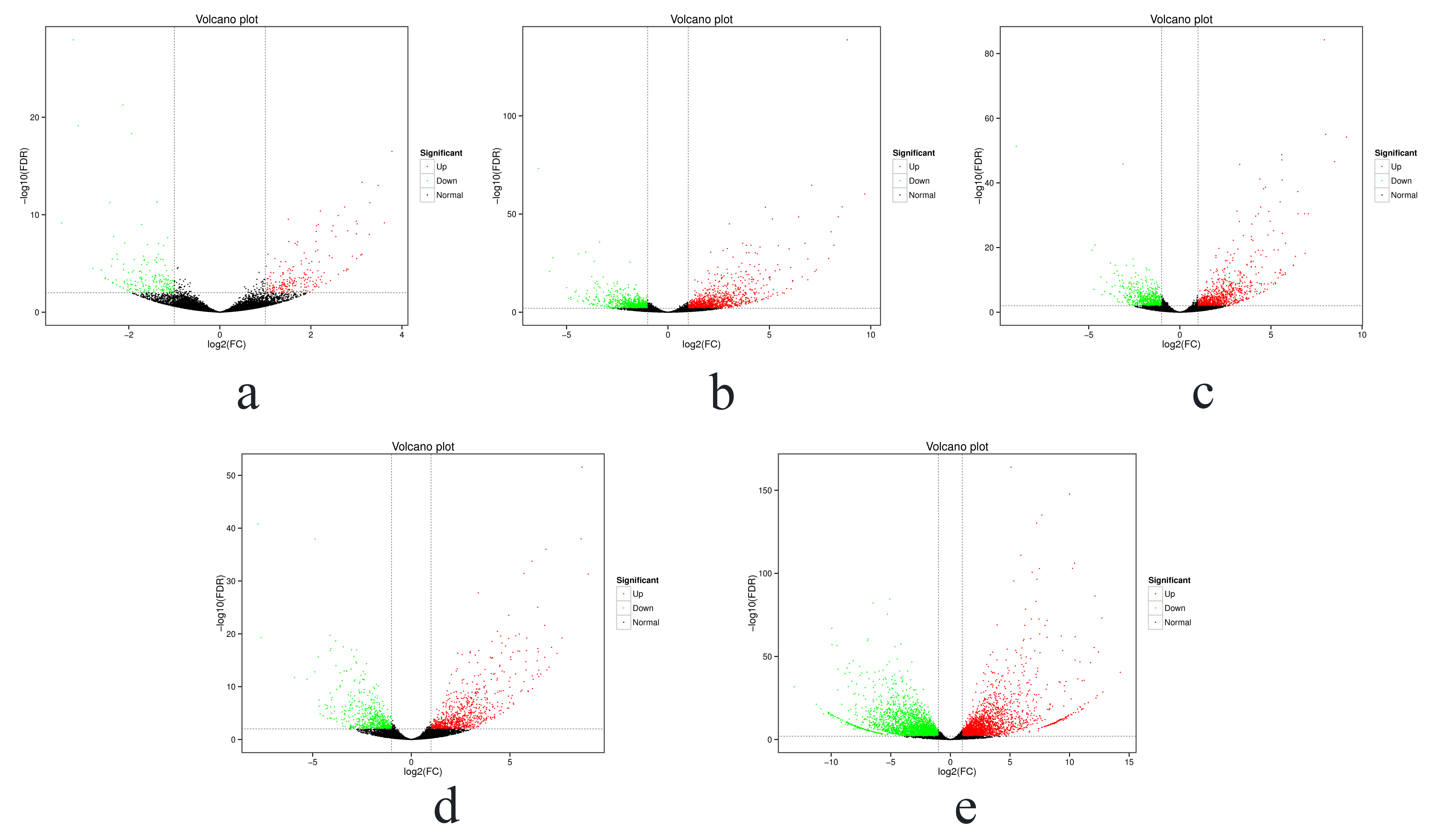

Supplement: Supplementary file 1 [file genes-11-01228-s001.zip › Supplementary Files/Figure S1.png]

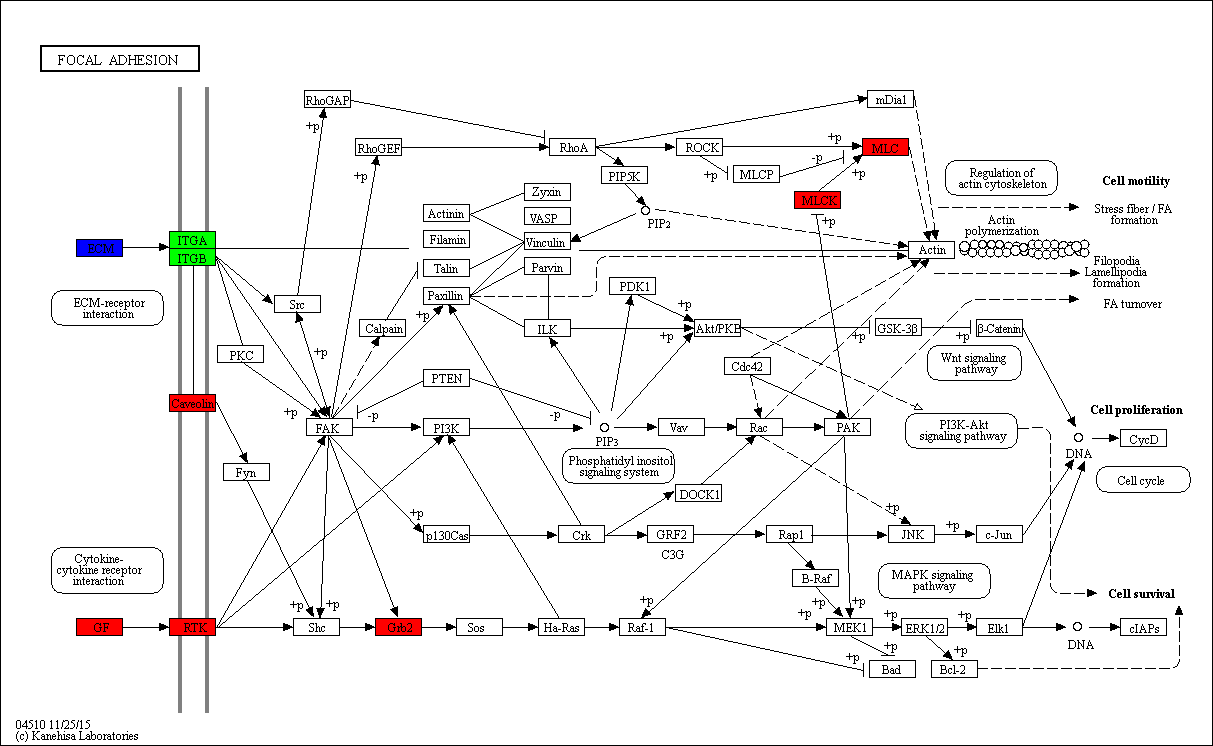

Supplement: Supplementary file 1 [file genes-11-01228-s001.zip › Supplementary Files/Figure S10.png]

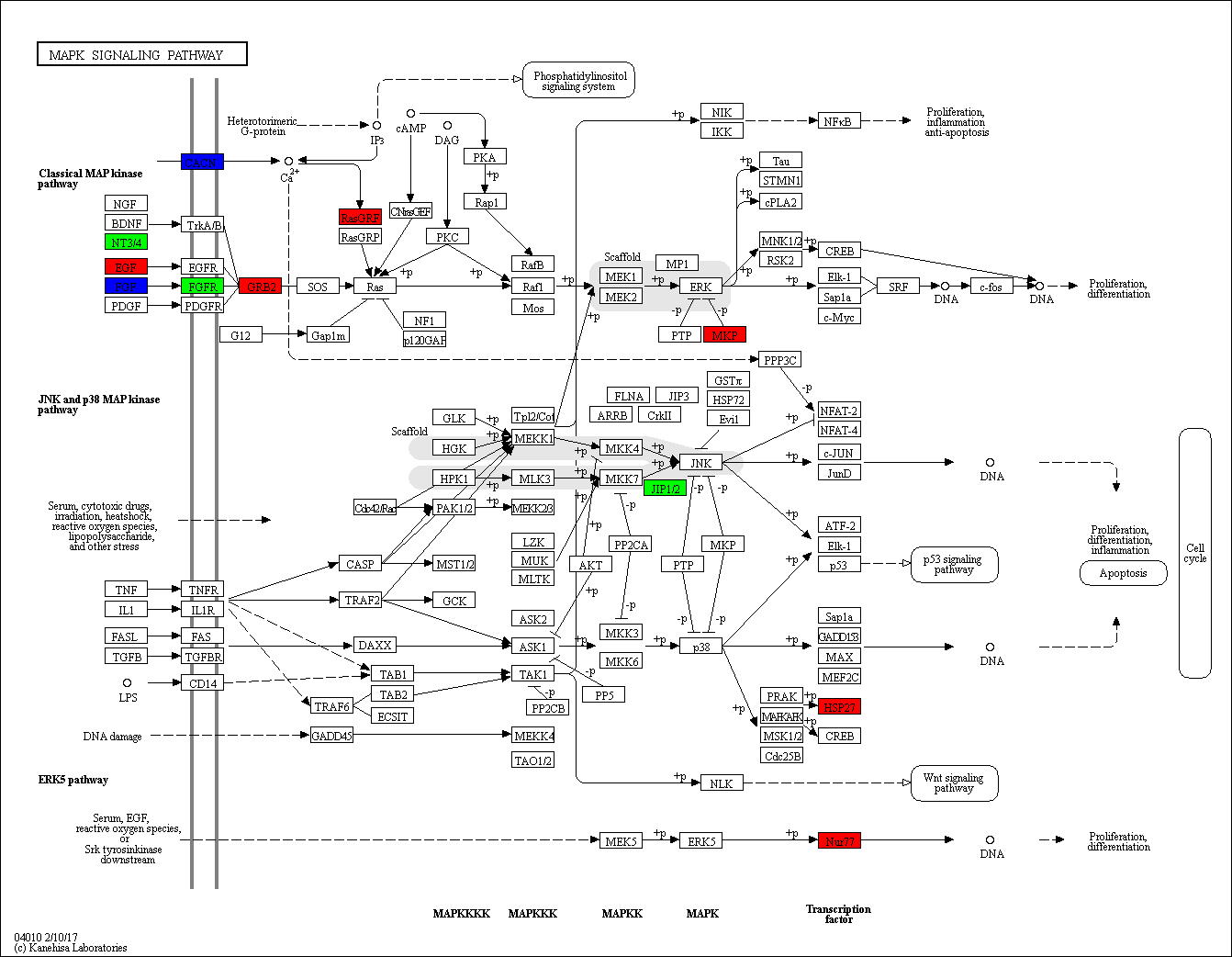

Supplement: Supplementary file 1 [file genes-11-01228-s001.zip › Supplementary Files/Figure S11.png]

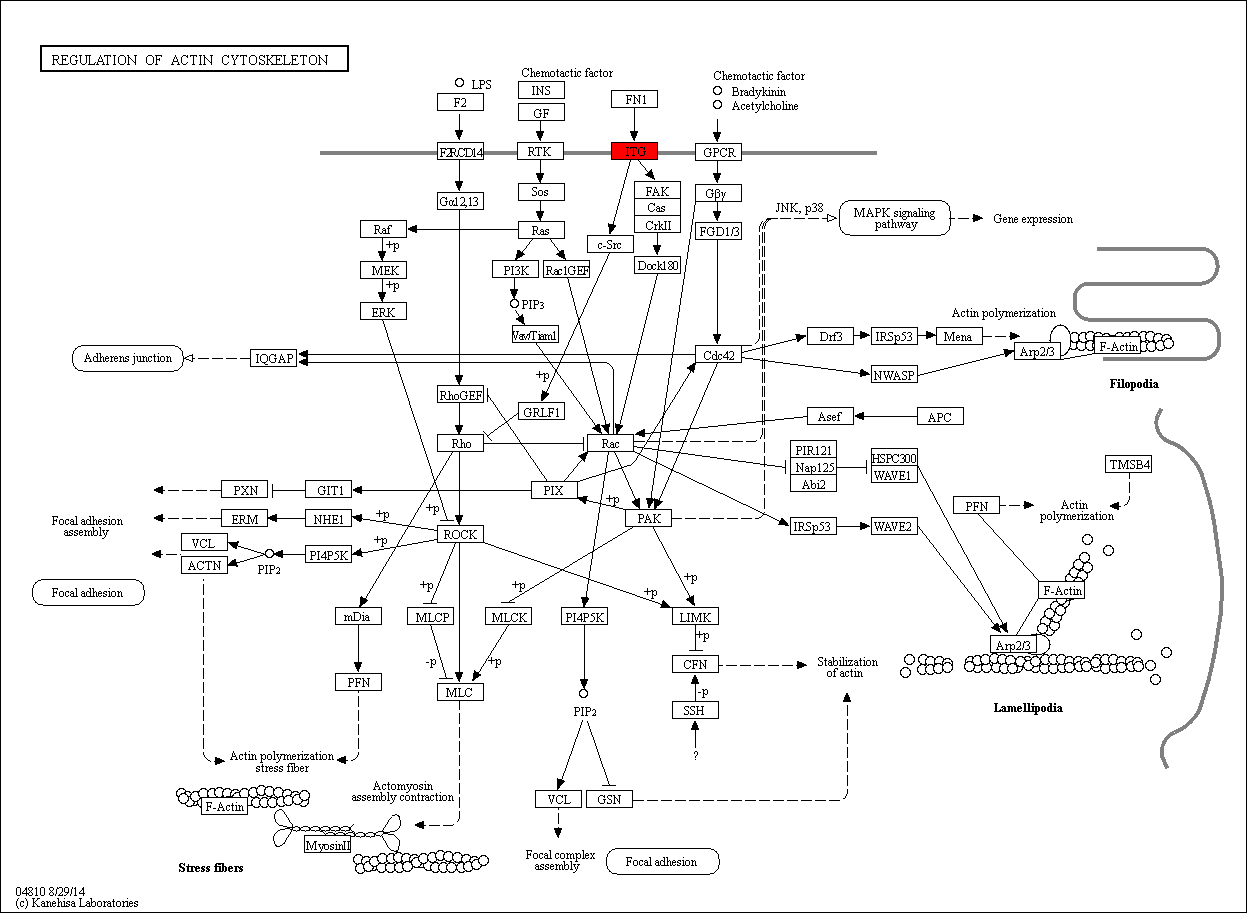

Supplement: Supplementary file 1 [file genes-11-01228-s001.zip › Supplementary Files/Figure S12.png]

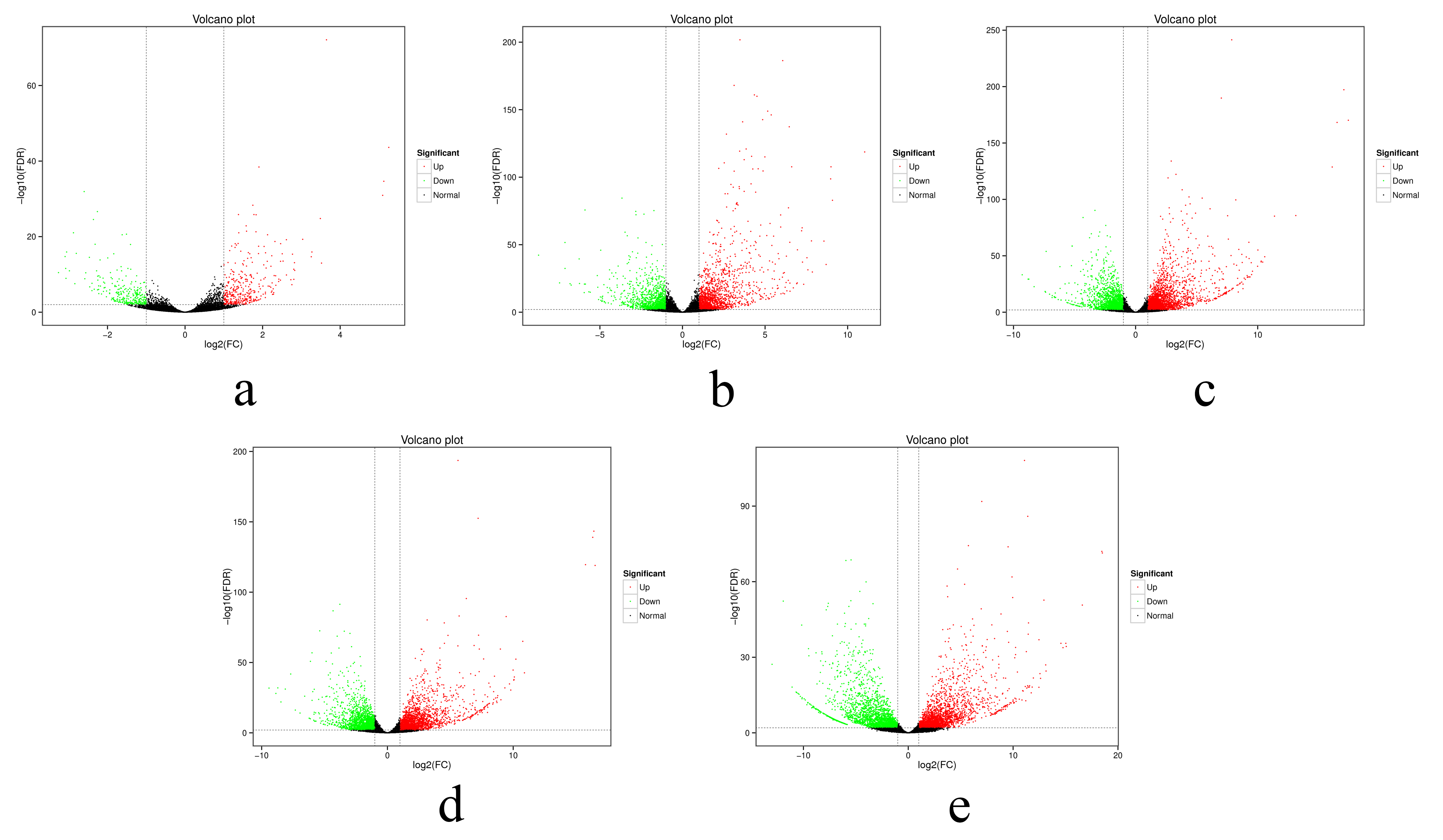

Supplement: Supplementary file 1 [file genes-11-01228-s001.zip › Supplementary Files/Figure S2.png]

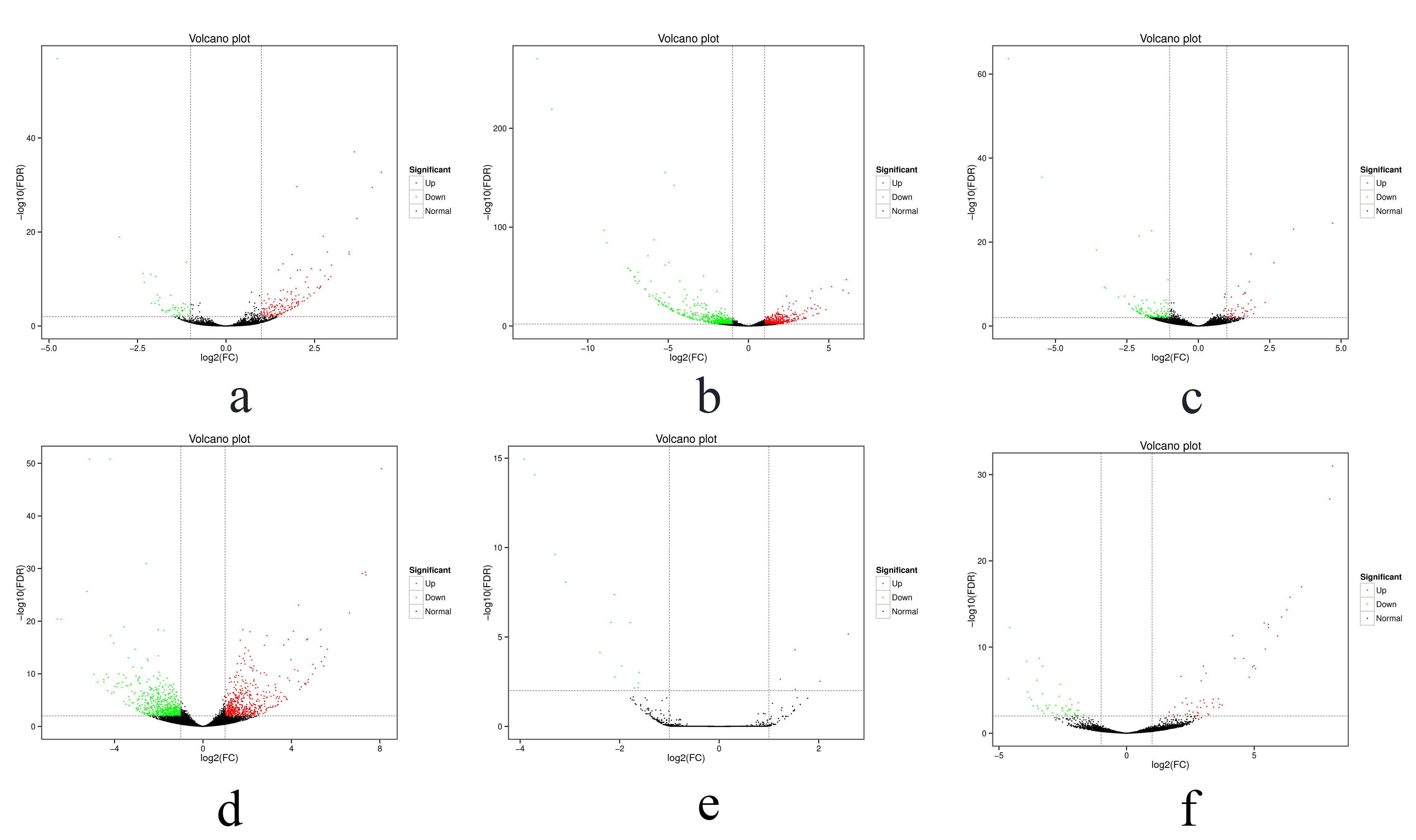

Supplement: Supplementary file 1 [file genes-11-01228-s001.zip › Supplementary Files/Figure S3.png]

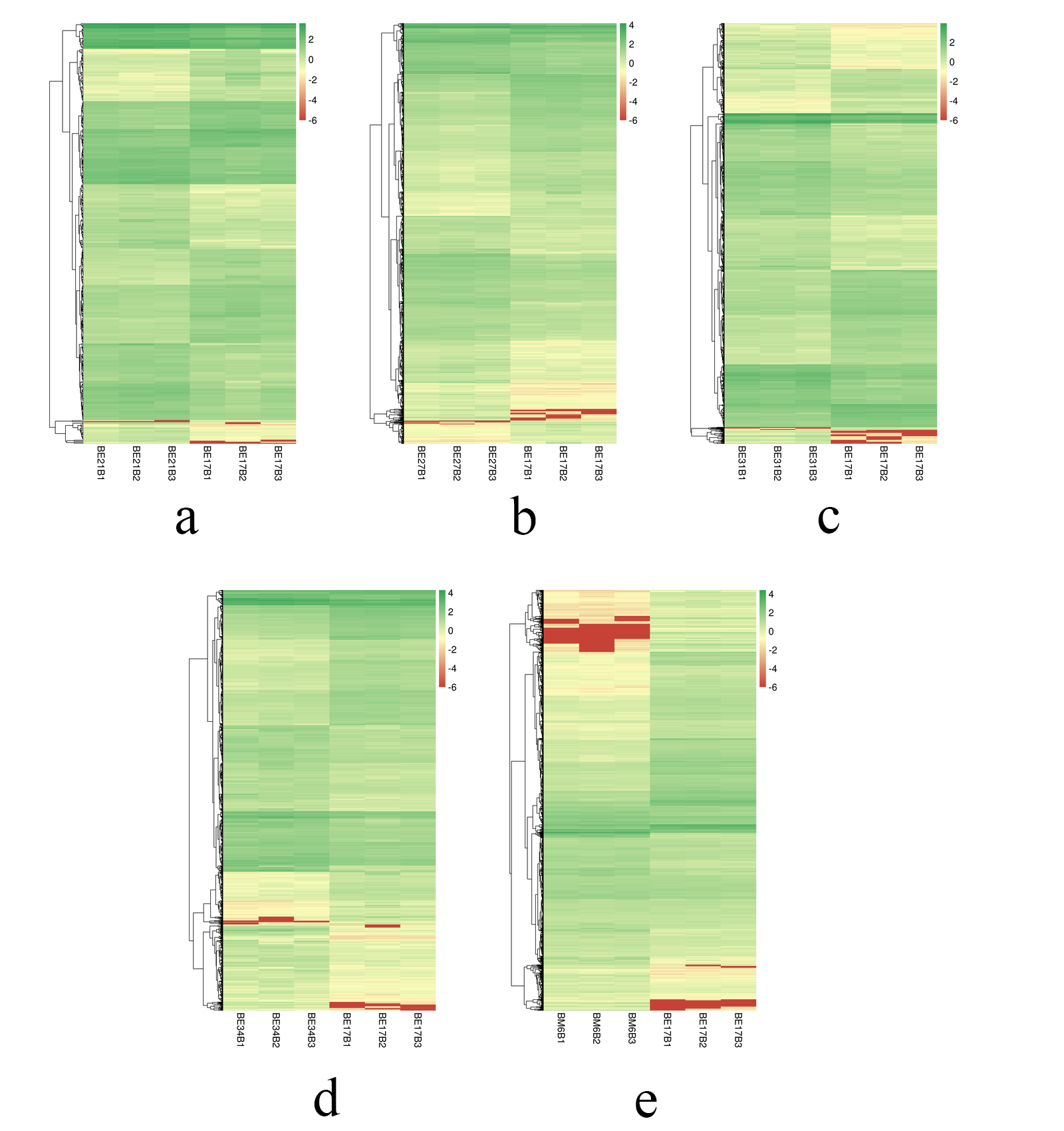

Supplement: Supplementary file 1 [file genes-11-01228-s001.zip › Supplementary Files/Figure S4.png]

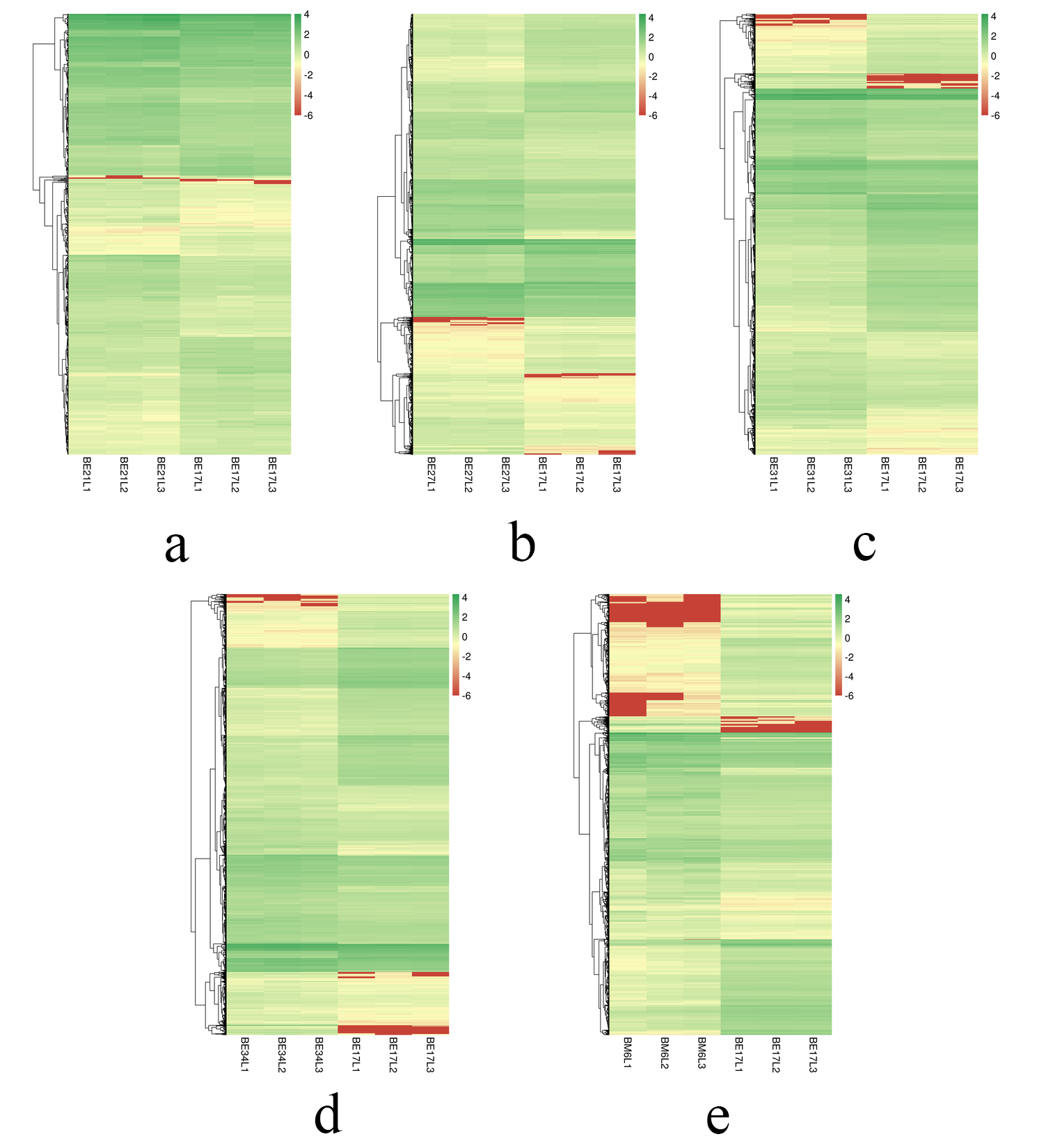

Supplement: Supplementary file 1 [file genes-11-01228-s001.zip › Supplementary Files/Figure S5.png]

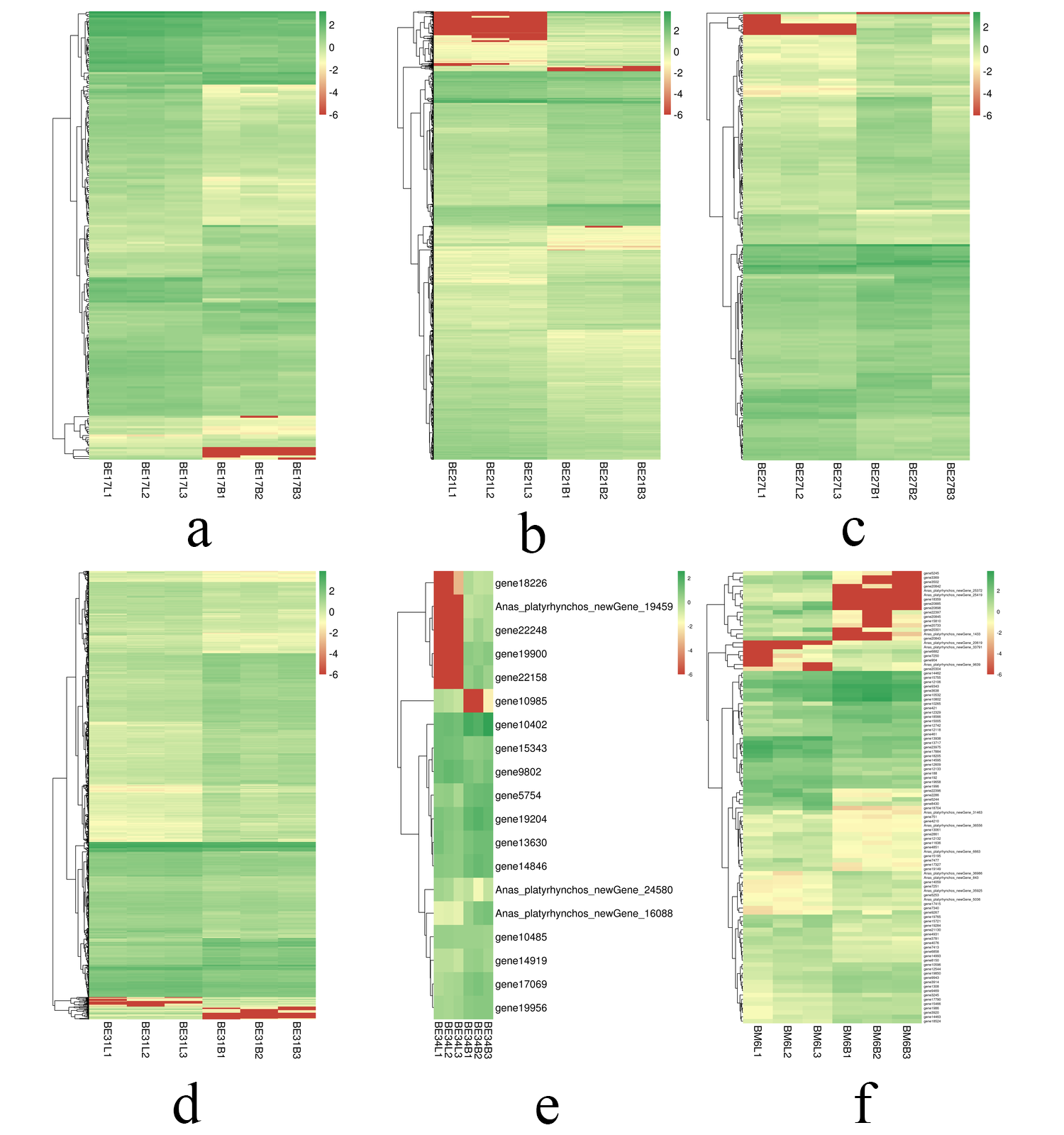

Supplement: Supplementary file 1 [file genes-11-01228-s001.zip › Supplementary Files/Figure S6.png]

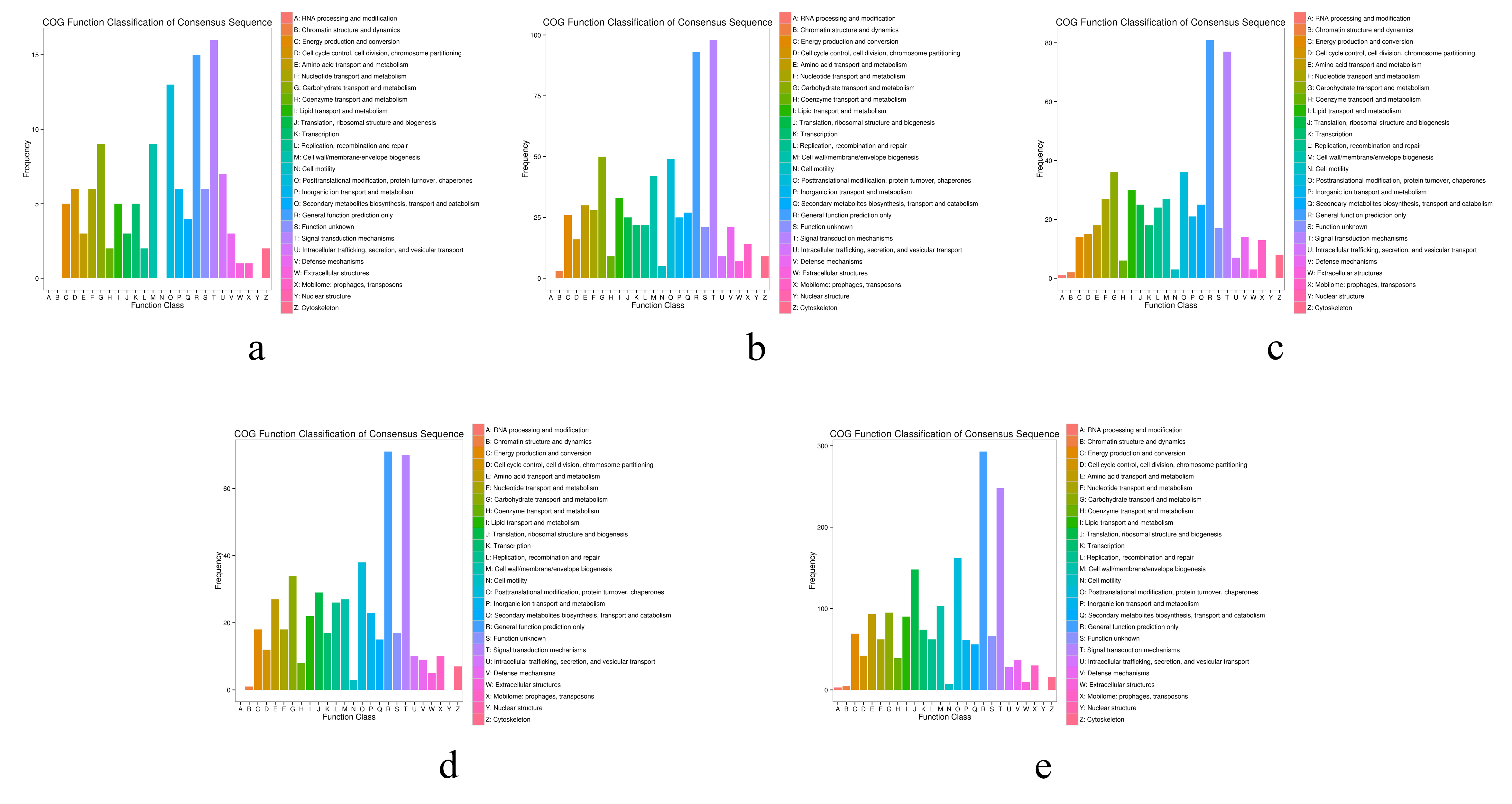

Supplement: Supplementary file 1 [file genes-11-01228-s001.zip › Supplementary Files/Figure S7.png]

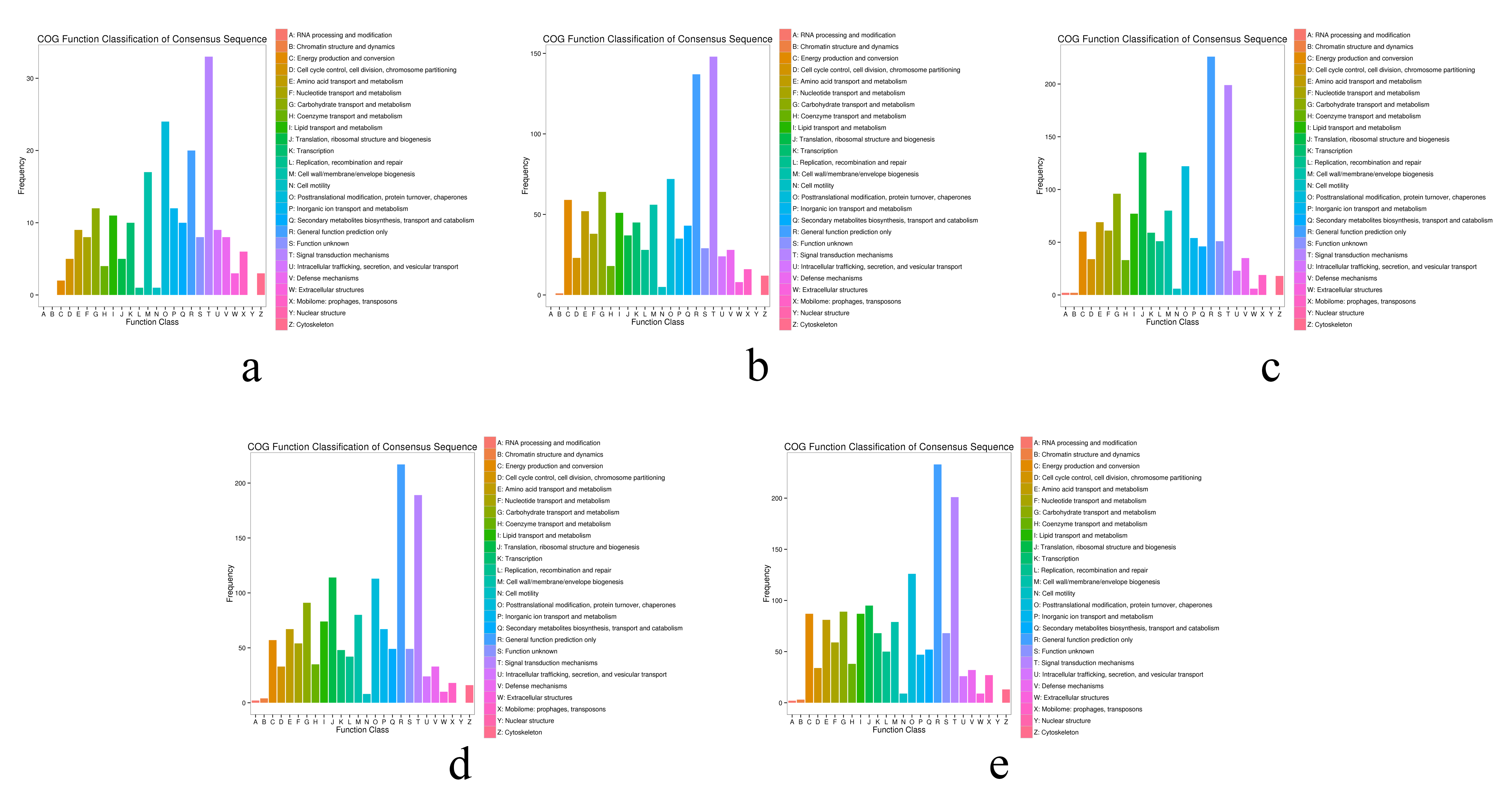

Supplement: Supplementary file 1 [file genes-11-01228-s001.zip › Supplementary Files/Figure S8.png]

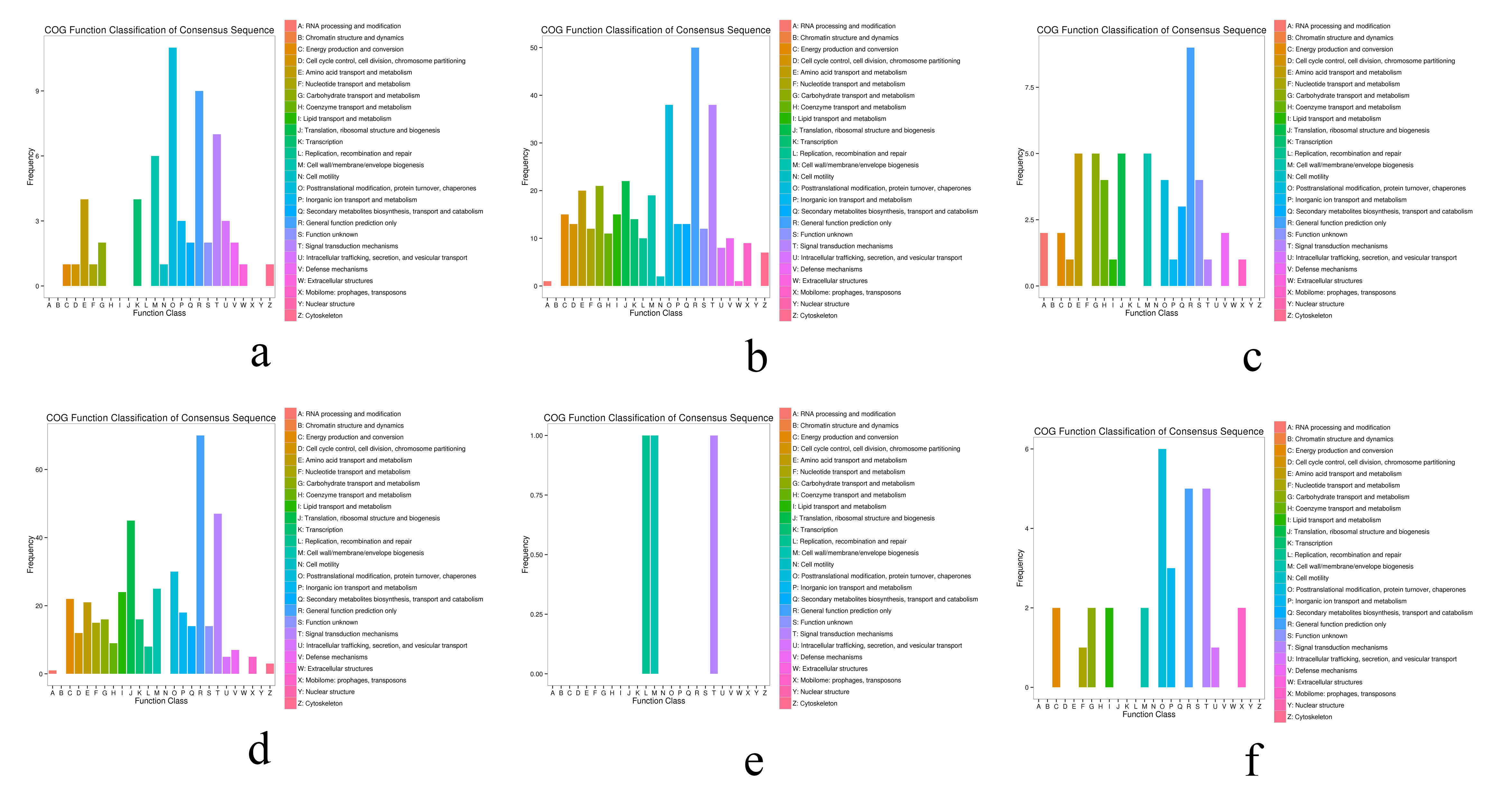

Supplement: Supplementary file 1 [file genes-11-01228-s001.zip › Supplementary Files/Figure S9.png]
